# Whole genome sequencing of experimental hybrids supports meiosis-like sexual recombination in *Leishmania*

**DOI:** 10.1371/journal.pgen.1008042

**Published:** 2019-05-15

**Authors:** Ehud Inbar, Jahangheer Shaik, Stefano A. Iantorno, Audrey Romano, Chukwunonso O. Nzelu, Katherine Owens, Mandy J. Sanders, Deborah Dobson, James A. Cotton, Michael E. Grigg, Stephen M. Beverley, David Sacks

**Affiliations:** 1 Laboratory of Parasitic Diseases, National Institutes of Allergy and Infectious Diseases, National Institutes of Health, Bethesda, Maryland, United States of America; 2 Department of Molecular Microbiology, Washington University School of Medicine in St Louis, St Louis, Missouri, United States of America; 3 Wellcome Sanger Institute, Wellcome Genome Campus, Hinxton, Cambridge, United Kingdom; Seattle Biomedical Research Institute, UNITED STATES

## Abstract

Hybrid genotypes have been repeatedly described among natural isolates of *Leishmania*, and the recovery of experimental hybrids from sand flies co-infected with different strains or species of *Leishmania* has formally demonstrated that members of the genus possess the machinery for genetic exchange. As neither gamete stages nor cell fusion events have been directly observed during parasite development in the vector, we have relied on a classical genetic analysis to determine if *Leishmania* has a true sexual cycle. Here, we used whole genome sequencing to follow the chromosomal inheritance patterns of experimental hybrids generated within and between different strains of *L*. *major* and *L*. *infantum*. We also generated and sequenced the first experimental hybrids in *L*. *tropica*. We found that in each case the parental somy and allele contributions matched the inheritance patterns expected under meiosis 97–99% of the time. The hybrids were equivalent to F1 progeny, heterozygous throughout most of the genome for the markers that were homozygous and different between the parents. Rare, non-Mendelian patterns of chromosomal inheritance were observed, including a gain or loss of somy, and loss of heterozygosity, that likely arose during meiosis or during mitotic divisions of the progeny clones in the fly or culture. While the interspecies hybrids appeared to be sterile, the intraspecies hybrids were able to produce backcross and outcross progeny. Analysis of 5 backcross and outcross progeny clones generated from an *L*. *major* F1 hybrid, as well as 17 progeny clones generated from backcrosses involving a natural hybrid of *L*. *tropica*, revealed genome wide patterns of recombination, demonstrating that classical crossing over occurs at meiosis, and allowed us to construct the first physical and genetic maps in *Leishmania*. Altogether, the findings provide strong evidence for meiosis-like sexual recombination in *Leishmania*, presenting clear opportunities for forward genetic analysis and positional cloning of important genes.

## Author summary

*Leishmania* promastigotes are able to undergo genetic exchange during their growth and development in the sand fly vector, however, it is still not known if they have a true sexual cycle involving meiosis. Here, we used whole genome sequencing to follow the chromosomal inheritance patterns of 44 experimental hybrids generated between different strains of *L*. *major*, *L*. *infantum*, and *L*. *tropica*. In almost every case the number of chromosomes and the allele contributions from each parent matched the inheritance patterns expected under meiosis. Rare instances of hybrid chromosomes that did not fit Mendelian expectations were observed, including gain or loss of somy, and loss of heterozygosity. Strong evidence for a meiotic-like process was also obtained from the genome wide patterns of recombination observed in the offspring generated from backcrosses involving an experimental or natural hybrid, consistent with crossing over occurring between homologous chromosomes during meiosis. The frequency and position of the recombination breakpoints observed on each chromosome allowed us to construct the first physical and genetic maps in *Leishmania*. The results demonstrate that forward genetic approaches are possible for positional cloning of important genes.

## Introduction

Protozoan parasites of the genus *Leishmania* present a remarkable epidemiologic and clinical diversity, producing a spectrum of human and veterinary diseases ranging from localized, self-limiting cutaneous lesions, to more chronic and destructive mucocutaneous involvement, to disseminating, visceral infection that is fatal in the absence of treatment. *Leishmania* have a dimorphic life cycle consisting of extracellular promastigotes that multiply within the alimentary tract of the sand fly vector, and intracellular amastigotes that multiply within host mononuclear cells. The diversity of clinical outcomes, as well as reservoir host range and vector species compatibilities, have distinct parasite species associations, with over 20 species associated with human infections. The origins of this diversity, whether by gradual accumulation of mutations through mitotic cell division, and/or by sexual recombination producing admixtures of divergent genomes, remain a matter of considerable debate [1].

Hybridization, defined as reproduction between members of genetically distinct populations and producing offspring of mixed ancestry [2], is common in nature and has wide-ranging effects on speciation and the evolution of populations. The isolation of *Leishmania* strains that have been characterized as hybrids is by now well described. Multi locus genotyping using a variety of techniques identified hybrids between closely related New World species [3–7], between closely related Old World species [8–10], and between two very divergent species, *L*. *infantum* and *L*. *major* [11]. Using more discriminatory genotyping approaches, mainly whole genome sequencing, natural hybridization has also been reported at the intraspecific level for *L*. *infantum*, *L*. *donovani*, and *L*. *tropica* [12–14]. Some *L*. *tropica* strains in particular show high levels of allelic diversity and heterozygosity consistent with full genome-hybridization due to natural outcrossing. Experimentally, we and others have demonstrated that inter- and intraspecific hybrids can be generated in the sand fly vector, formally demonstrating that promastigote stages of *Leishmania* possess the machinery for genetic exchange [15–18]. Using pairwise combinations of parental lines expressing distinct drug resistant markers, double drug resistant lines could be recovered from sand flies co-infected with different strains of *L*. *major*, or with *L*. *major* and *L*. *infantum*, that in every case appeared to be full genomic hybrids based on their bi-parental inheritance of a limited number of allelic markers distributed across the nuclear genome. The majority of the experimental hybrids were close to diploid, though triploid and tetraploid offspring were also observed. Mating competency was confined to promastigote stages developing in the fly, and both Old and New World vector species could support hybrid formation. Based on the experimental outcrosses performed so far in which only a low frequency of co-infected flies yielded hybrids (2–20%), mating must be considered a non-obligatory part of the parasite life-cycle. Overall, the current debate regarding *Leishmania* reproductive strategies reflects mainly the mode of genetic exchange, its frequency and impact on population structure, not whether or not it occurs [19, 20].

Among kinetoplastid protists, the most well studied mating system is that of *Trypanosoma brucei*, for which a meiotic process is well supported based on the identification of a haploid parasite stage in the vector [21], and on the patterns of allele inheritance and recombination observed in experimental hybrids [22, 23]. While genome hybridization is one of the signatures of meiosis, it can also be explained by a parasexual process, as observed in some fungi [24] and proposed for *Trypanosoma cruzi* [25] and *Leishmania* [26], involving fusion of cells from both parents with generation of a transient polyploid state, followed by chromosome shuffling and random loss. True sex, incorporating meiosis with generation of haploid gametes or gamete-like cells, cell fusion or syngamy, and fusion of haploid nuclei, has not been directly observed in *Leishmania*, although in vitro cell fusion events were recorded in 1990 for two species, *L*. *infantum* and *L*. *tropica* [27]. Each of these processes, if they occur at all, may be difficult to detect because sex does not appear to be an obligatory stage of the life cycle, there is no obvious sexual dimorphism, and the mating competent forms are so far confined to promastigote stages developing in vivo, i.e. the sand fly midgut. While sex might be inferred from the presence and expression of meiotic gene orthologues in *Leishmania* [28], these orthologues can have other functions and are known to be maintained even in asexual species [29]. We have therefore turned to a genetic analysis of experimental hybrids, for which chromosome inheritance patterns expected under meiosis might be revealed, including balanced parental contributions, and recombination between homologous chromosomes. Importantly, in so far as variation in chromosome copy number is thought to be a constitutive, well tolerated, and potentially adaptive mechanism in *Leishmania* [30–32], then analysis of the genome structures of experimental hybrids can also reveal somy inheritance patterns and the extent to which genome hybridization is a source of aneuploid variation.

In the current studies of 3 different species of *Leishmania*, including *L*. *major*, *L*. *infantum*, and *L*. *tropica*, we have used whole genome sequencing to reveal the genome structures, chromosome inheritance patterns, and recombination events present in experimental intra- and interspecies hybrids. The highly predictable somy and allele inheritance patterns, and especially the genome wide recombinations observed in backcrosses involving experimental and natural hybrids, provide strong evidence for a meiotic-like sexual cycle in *Leishmania*.

## Results

### Whole genome sequencing of intraspecies hybrids in *L*. *major*

We have previously described the recovery of hybrids from *P*. *duboscqi* and *Lu*. *longipalpis* sand flies co-infected with different pairwise combinations of *L*. *major* strains originating from across the geographic range of this species [15, 16]. For the whole genome sequencing analysis, we selected for comparison all of the hybrids previously described, plus two additional hybrids (fl6b and fl5b in Table 1A), that were generated between LmFV1/SAT, originating in Israel, and LmLV39/HYG, originating from southern Russia. We further confined the initial analysis to the hybrids that were generated in *P*. *duboscqi*, a natural vector of *L*. *major* transmission, and that had an approximate 2n DNA content, for which assessing parental inheritance is more tractable compared to the polyploid hybrids. The parental and 16 progeny clone sequences were aligned to *L*. *major Friedlin FV1* genome Version 6 (LmjFV1.06)(http://tritrypdb.org) using Novoalign (http://www.novocraft.com/), and yielded an average 60x coverage per sample. The mapped reads were processed to obtain total read depth, reference, and alternate allele frequencies using AGELESS software (http://ageless.sourceforge.net/). The analysis identified 76103 homozygous SNPs in the LmLV39/HYG parent in comparison to LmFV1/SAT, or 0.11 SNPs/Kb, and only a relatively low number (3573) of heterozygous SNPs (Table 1A). By contrast, each hybrid possessed a high number of heterozygous SNPs (72,449–77,069; Table 1A), reflecting their bi-allelic inheritance of the approximate 76,000 SNPs that were homozygous and different between the parents.

**Table 1:** Heterozygous and homozygous SNPs identified in parents and hybrids.

Sample	Heterozygous SNPs (SNPs/Kb)	Homozygous SNPs (SNPs/Kb)	Total SNPs (SNPs/Kb)	Origin
**A** **LmFV1/SAT vs**	**3573 (0.11)**	**76103 (2.37)**	**79676 (2.49)**	**Israel**
**LmLV39/HYG**				**Russia**
1.10.b1	77019 (2.4)	419 (0.01)	77438 (2.4)	Hybrid
1.10.d9	76340 (2.4)	1196 (0.04)	77536 (2.4)	“
1.16.a1	76859 (2.4)	372 (0.01)	77231 (2.4)	“
4.3.a12	76942 (2.4)	411 (0.01)	77353 (2.4)	“
4.3.g12	76693 (2.4)	399 (0.01)	77092 (2.4)	“
4.7.a3	76856 (2.4)	424 (0.01)	77280 (2.4)	“
5.12.d9	76113 (2.4)	1235 (0.04)	77348 (2.4)	“
5.12.f11	77069 (2.4)	396 (0.01)	77465 (2.4)	“
5.22.a10	74088 (2.3)	353 (0.01)	74441 (2.3)	“
6.14.f9	72449 (2.2)	439 (0.01)	72888 (2.2)	“
fl13d8	76381 (2.4)	373 (0.01)	76754 (2.4)	“
fl13e3	76715 (2.4)	402 (0.01)	77117 (2.4)	“
fl13e6	74226 (2.3)	420 (0.01)	74646 (2.3)	“
fl13f8	75604 (2.4)	474 (0.01)	76078 (2.4)	“
fl6b	76918 (2.4)	315 (0.01)	77233 (2.4)	“
fl5b	76365 (2.4)	355 (0.01)	76720 (2.4)	“
**B** **LiL/HYG vs LmFV1/SAT**	**12256 (0.38)**	**674569 (21.0)**	**687923 (21.5)**	**Spain Israel**
LimH2	674613 (21.0)	6051 (0.19)	680664 21.2)	Hybrid
LimH4	661722 (21.0)	5801 (0.19)	667523 (20.8)	“
LimH6	671313 (21.0)	6233 (0.19)	677546 (21.1)	“
LimH7	668230 (21.0)	6059 (0.19)	674289 (21.1)	“
LimH11	674403 (21.1)	6226 (0.19)	680669 (21.2)	“
**C** **LtL747/HYG vs LtMA37/NEO**	**57569 (0.18)**	**157085 (4.8)**	**162841 (5.2)**	**Israel Jordan**
LtHLMA1a	166612 (5.2)	2673 (0.08)	169285 (5.3)	Hybrid
LtHLMA1b	162823 (5.1)	2654 (0.08)	165477 (5.2)	“
LtHLMA2a	155853 (4.9)	2780 (0.09)	158633 (5.0)	“
LtHLMA2b	155485 (4.8)	3153 (0.1)	158638 (5.0)	“
LtHLMA3a	157649 (4.9)	2883 (0.09)	160532 (5.0)	“
LtHLMA3b	159932 (5.0)	2541 (0.08)	162473 (5.1)	“
LtHLMA4a	157428 (4.9)	2353 (0.07)	159781 (5.0)	“
LtHLMA4b	155428 (4.9)	2753 (0.09)	158181 (5.0)	“
LtHLMA5a	156294 (4.9)	2775 (0.09)	159069 (5.0)	“
LtHLMA5b	156112 (4.9)	2287 (0.07)	158399 (5.0)	“
**D**				
**LtL747/HYG vs LtKub/SAT**	**231919 (7.2)**	**153878 (4.8)**	**385797 (12.0)**	**Israel Syria**
LtHKLA2	276581 (8.7)	108919 (3.4)	385500 (12.0)	Hybrid
LtHKLA3	270815 (8.5)	114991 (3.6)	385806 (12.0)	“
LtHKLA4	275474 (8.6)	110059 (3.4)	385533 (12.0)	“
LtHKLA5	260101 (8.1)	125074 (3.9)	385175 (12.0)	“
LtHKLA7	265181 (8.3)	119833 (3.7)	385014 (12.0)	“
LtHKLA8	277260 (8.7)	108566 (3.4)	385826 (12.0)	“
LtHLKLA9	262049 (8.2)	123487 (3.9)	385536 (12.0)	“
LtHKLA10	257521 (8.0)	128142 (4.0)	385663 (12.0)	“
**E**	
**LtMA37/NEO vs LtKub/SAT**	**216390 (6.8)**	**23775 (0.74)**	**240165 (7.5)**			**Jordan Syria**
LtHKMA1	137512 (4.3)	103393 (3.2)	240905 (7.5)			Hybrid
LtHKMA2	127450 (4.0)	113253 (3.5)	240703 (7.5)			“
LtHKMA3	145872 (4.6)	94922 (3.0)	240794 (7.5)			“
LtHKMA4	141061 (4.4)	99682 (3.1)	240743 (7.5)			“
LtHKMA5	148304 (4.6)	92550 (2.9)	240854 (7.5)			“
LtHKMA6	134767 (4.2)	106080 (3.3)	240847 (7.5)			“
LtHKMA10	143621 (4.5)	97442 (3.0)	241063 (7.5)			“
LtHKMA11	144227 (4.5)	96543 (3.0)	240770 (7.5)			“
LtHKMA12	136050 (4.3)	104632 (3.3)	240682 (7.5)			“

**Fig 1:**
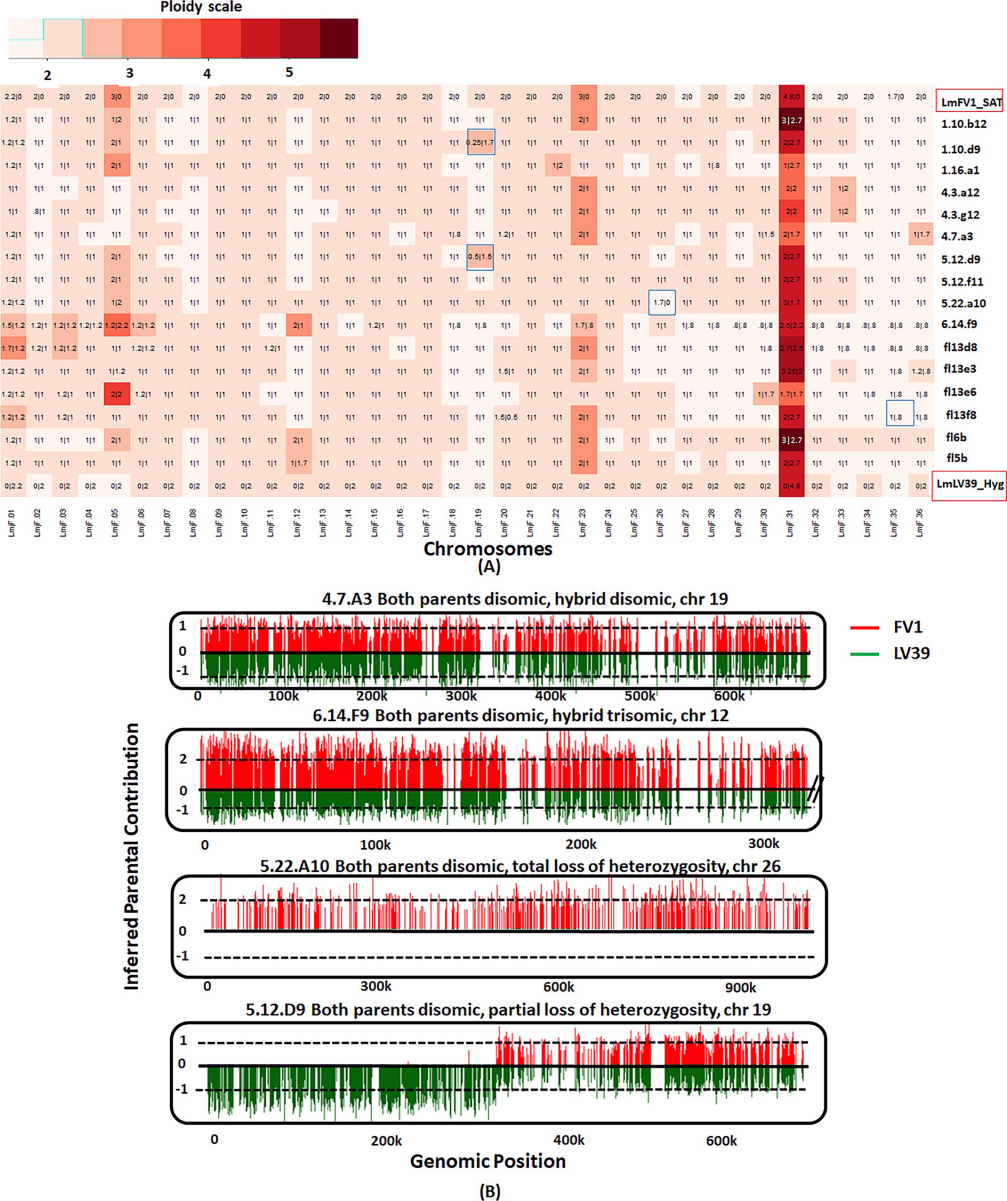
Somies and parental contributions in *L*. *major* intraspecies hybrids. **A)** The hybrids selected for analysis were generated between LmFV1/SAT and LmLV39/HYG in *P*. *duboscqi* flies, and were all close to 2n. The counts of read alignments across the genome were translated into somy values (see methods) which were plotted as a heatmap, rounded off to the closest 0.5 value. Overlaid are the parental inheritance values in the format LmFV1-SAT/LmLV39-HYG, scaled to nearest 0.1 value. The blue boxes indicate chromosomes for which one of the parents contributes partially (partial LOH) or not at all (total LOH). **B)** Bottle brush plots of representative chromosomes in *L*. *major* hybrids, with selected chromosomes showing either balanced contribution of parental alleles, gain of somy, complete or partial LOH. Homozygous parental SNP differences were identified and SNPs mapping to the LmFV1/SAT are shown in red and those mapping to LmLV39/HYG are shown in green. The vertical distance corresponds to the inferred allelic depth, normalized across the entire genome, which was assigned an average somy of 2.

The somy values of the parents and progeny clones were rounded off to the closest 0.5 value and depicted using a heatmap (Fig 1A). The actual somy values are not absolute integers (S1 Table), which may be attributed to the tendency of *Leishmania* chromosomes to show somy differences amongst cells in culture, referred to as mosaic aneuploidy [33]. Overlaid on the heatmap are the proportionate values for each parental contribution, rounded off to the closest 0.1. The profiles indicate that both parents were mostly disomic, with the exception of chromosome 31, which was pentasomic in both the parents, and chromosomes 5 and 23, which were trisomic in LmFV1/SAT. Of the chromosomes that were disomic in both parents (16x33 = 528 chromosome copies), 98% were disomic in the hybrids, and of these, 99% showed a variant allele frequency of roughly 0.5, having inherited an equal contribution from both parents, consistent with a meiosis-like process. For the two chromosomes that were trisomic in the LmFV1/SAT parent (16x2 = 32 chromosomes), we found disomic and trisomic hybrid chromosomes 41% and 53% of the time, respectively, close to expected frequencies for a meiotic process in which gametes have a roughly 50% chance of receiving either one or two copies of each trisomic chromosome. Of the trisomic chromosomes, all but 2 inherited their extra copy from the LmFV1/SAT parent, as expected. For chromosome 31, the hybrid somies ranged from 4–5, with each parent contributing two copies in most cases.

The main exceptions to the expected chromosome inheritance patterns were the 2% of chromosomes in which a new trisomic chromosome was contributed from a disomic parent (12/576 = 2%), and the 0.7% of the chromosomes for which a partial or total loss of heterozygosity (LOH) was inferred (highlighted by blue boxes). The LOH events were visualized using bottle brush plots in which the allele count at each SNP position is displayed. Bottle brush plots of representative chromosomes from hybrids showing either balanced contribution of parental alleles, gain of somy, or complete or partial LOH are shown in Fig 1B. Each partial LOH is thought to have arisen from a single crossover event that likely occurred following meiosis.

#### Whole genome sequencing of interspecies hybrids

We have also previously described experimental cross-species hybridization between a visceral strain of *L*. *infantum* LLM-320 originating from Spain (LiL/HYG), and the cutaneous *L*. *major* strain from Israel (LmFV1/SAT) [17]. The co-infections were carried out in *Lu*. *longipalpis* sand flies, which is a natural vector of *L*. *infantum*, as well as a permissive vector in the laboratory for *L*. *major*. All of the progeny clones previously reported, including 5 diploid, 4 triploid, and 1 tetraploid hybrid, and derived from 10 different flies, were subjected to whole genome sequencing to further characterize the hybrid genomes. The LiL/HYG parent had 687923 SNPs, or 21 SNPs/Kb, that were homozygous different from LmFV1/SAT, and 12256 heterozygous SNPs (Table 1B). The interspecies mating produced hybrids that in each case were heterozygous at positions where each of the parents was homozygous for a different allele, resulting in a high number of heterozygous SNPs (661772–674613) (Table 1B).

**Fig 2:**
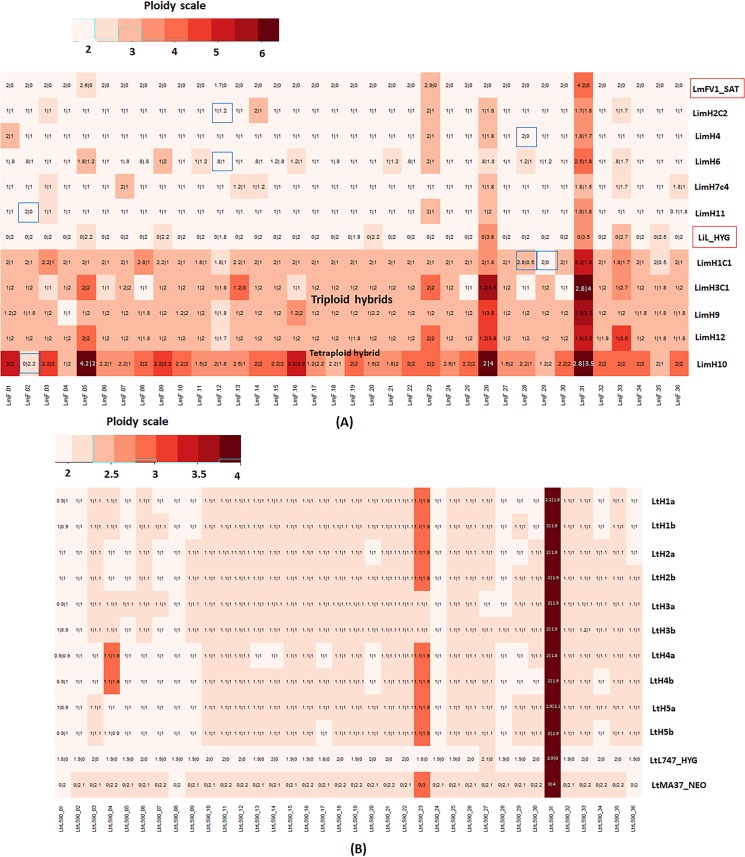
Somies and parental contributions in interspecies and *L*. *tropica* intraspecies hybrids. **A)** The interspecies hybrids were all generated between LmFV1/SAT and LiL/HYG in *Lu*. *longipalpis* flies, and include five 2n, four 3n, and one 4n hybrid. On the heatmap, the somy values were rounded off to the closest 0.5, and are overlaid with the parental inheritance values in the format LmFV1-SAT/LiL-HYG, rounded off to the nearest 0.1. The chromosomes with partial or total loss of heterozygosity are indicated by the blue boxes. **B)** Somies and parental contributions in *L*. *tropica* hybrids, showing 2 clones (a and b) from each of 5 different hybrid lines recovered from 5 different flies. The somy values are represented as a heatmap, rounded off to the nearest 0.25 value. Overlaid are the parental inheritance values in the format LtL747-HYG/LtMA37-NEO, rounded off to the nearest 0.1 value. Most hybrid chromosomes have balanced contributions from each parent, but in two clones, extra contributions by one parent are observed. The replicate clones have identical genotypes in each case.

The somy profile of the LiL/HYG parent shows that most chromosomes had somy close to 2, while chromosome 33 was trisomic, and chromosomes 26 and 31 were tetrasomic (S1 Table, Fig 2A). For the approximate 2n hybrids, when both the parents were disomic (5x31 = 155 chromosomes), the hybrid chromosomes were also disomic 97% of the time, with roughly equivalent contribution from each parent. Unexpected trisomy was observed 3% of the time, with the LmFV1/HYG parent contributing the additional chromosome in each case. For the chromosomes that were trisomic in one or the other parent (5 x3 = 15 chromosomes), the hybrid chromosomes in the 2n hybrids were approximately disomic 47% of the time and trisomic 53% of the time, with the extra chromosome always contributed by the trisomic parent, as expected under a meiotic process in which the resulting gametes will have a roughly equal chance of inheriting 1 or 2 copies of that chromosome. The chromosomes that were tetrasomic in one or both of the parents were always passed on in double copy from the respective parent. For the 4 hybrids that were close to 3n, 94% of the chromosomes that were disomic in both parents, and all of the chromosomes that were trisomic in one or the other parent, possessed an extra copy that within each hybrid clone was always contributed by the same parent, consistent with a failure of reductional division during meiosis I, or its equivalent in *Leishmania*, in that parent. The extra genome was contributed by LiL/HYG to three of the hybrids, and by LmFV1/SAT to one. The 4n hybrid (LimH10) showed a majority of the chromosomes to be tetrasomic with roughly equal contribution from both the parents. The chromosomes showing somies >4 reflected contributions from the parental non-disomic chromosomes, although unexpected extra contributions from either parent were also observed (e.g. chr 1 & 16). Finally, 7 chromosomes (1.9%) showed partial or total LOH, observed in both the diploid and polyploid hybrids (highlighted in blue boxes).

### Intraspecies mating and whole genome sequencing of experimental hybrids in *L*. *tropica*

*L*. *tropica* is the causative agent of zoonotic and anthroponotic cutaneous leishmaniasis (ACL), which is endemic throughout the Middle East and in some areas of Africa and the Indian sub-continent. Prior population genetic studies have identified discreet geographic regions where *L*. *tropica* isolates possess only low levels of heterozygous SNPs, and other regions where they display extensive heterozygosity, consistent with genetic exchange [13, 34, 35]. Experimentally, the mating competency of *L*. *tropica* strains has not been demonstrated, so far as we are aware. For the generation of experimental hybrids in *L*. *tropica*, we introduced stable drug resistance markers into two different strains, LtMA37/NEO originating from Jordan, and LtL747/HYG from Israel. In comparison to one another, these strains possess 157085 homozygous SNPs, or 4.9 SNPs/Kb, and only 5756 heterozygous SNPs (Table 1C). These strains were then used to co-infect *Lu*. *longipalpis*, a non-natural vector that is permissive to *L*. *tropica* development in our laboratory colonized flies. Out of a total of 143 co-infected flies, double drug resistant hybrids were recovered from 48 flies (34%), formally demonstrating the mating competency of members of this species. Ten of the 48 double drug resistant lines recovered from 10 different flies were cloned and tested by PCR to confirm inheritance of both parental antibiotic resistance markers (labeled as LtHLMA in S1 Fig).

Two diploid clones (a and b) generated from each of five different hybrid lines were selected for whole-genome sequencing. In each case the two clones presented nearly identical genotypes, and are likely derived from the same hybridization event. All of the hybrids were equivalent to F1 progeny, heterozygous at positions where each of the parents was homozygous for a different nucleotide, resulting in each of the hybrids possessing a high density of 155428–166612 heterozygous SNPs (4.9–5.2 SNPs/Kb) (Table 1C). In the somy analysis of the parents and each of the hybrid clones (Fig 2B), all appeared to be near-diploid. Of the chromosomes that were disomic in both parents (5x33 = 165), 99% were disomic in the hybrids, and all showed variant allele frequencies of close to 0.5. Chromosome 23 was trisomic in the LtMA37/NEO parent and all but one of the hybrids, with LtMA37/NEO contributing two copies to the trisomic chromosome, as expected. Chromosome 31 was tetrasomic in all samples, and showed variant allele frequencies of 0.5 in all of the hybrids. Thus, the parental somy contributions were non-random and 99% of the time met expectations of chromosomal segregation during a meiosis-like process. The exceptions were the hybrid clones LtHLM4a/b that were trisomic at chromosome 4 despite being disomic in both parents, possibly a result of chromosomal non-disjunction. No LOH was observed in any of the hybrids.

Altogether, the whole genome sequencing of the experimental hybrids generated within and between three different Old World *Leishmania* species demonstrate that the progeny clones are near full genomic hybrids, with highly predictable somy and allele inheritance patterns that are strongly consistent with a meiotic-like process. Rare instances of chromosomes showing a gain of somy or loss of heterozygosity were also observed.

### Backcross and outcross mating attempts involving an *L*. *major* F1 hybrid

To generate the first experimental backcross hybrids in *Leishmania* we chose a *L*. *major* hybrid, 1.16.A1, generated between LmFV1/SAT and LmLV39/HYG with ploidy close to 2n, and that demonstrated robust growth in culture and in flies. The mating studies involved co-infection of *P*. *duboscqi* flies with 1.16.A1 and *L*. *major* lines stably transfected with a third antibiotic resistance marker, blasticidin-S deaminase (BSD), and selection for midgut promastigotes that were doubly drug resistant to either SAT + BSD or to HYG + BSD. A series of 4 independent backcross experiments involving 1.16.A1 and LmFV1/BSD resulted in a low frequency of flies yielding hybrids (Table 2, expts 1–4). Of a total of 316 midguts from co-infected flies that could be evaluated for hybrid recovery, double drug resistant lines could be recovered from 2 flies (0.6%). PCR tests confirmed that both progeny clones had in fact inherited all three drug resistance markers (S2A Fig). In two experiments there were sufficient flies to include for comparison co-infections involving the parental lines, which in prior studies have produced an average of 11.3% hybrid recovery [15, 16]. In this case, co-infections with LmFV1/BSD and LmLV39/HYG produced recoverable hybrids in a total of 10 of 75 flies (13.3%). PCR tests confirmed their inheritance of both selectable markers (S2B Fig).

**Table 2:** Backcross/outcross matings in *P*. *duboscqi* involving an *L*. *major* intraspecies F1 hybrid.

Expt	Cross	Antibiotics	No. clean midguts^1^	No. hybrids recovered	% hybrid recovery
**1**	LmFV1-Bsd X 1.16.A1-Sat/Hyg **(NHxH)**^**2**^	Bsd-Sat/Hyg	61/52	0/1	1.6%
**2**	LmFV1-Bsd X 1.16.A1-Sat/Hyg **(NHxH)**	Bsd-Sat/Hyg	40/18	0/0	0%
**3**	LmFV1-Bsd X 1.16.A1-Sat/Hyg **(NHxH)**	Bsd-Sat/Hyg	119/62	1/0	0.8%
	LmFV1-Bsd x LmLv39 Hyg **(NHxNH)**	Bsd-Hyg	33	1	3.3%
**4**	LmFV1-Bsd X 1.16.A1-Sat/Hyg **(NHxH)**	Bsd-Sat/Hyg	96/65	0/0	0%
	LmFV1-Bsd x LmLv39-Hyg **(NHxNH)**	Bsd-Hyg	32	9	28%
**5**	LmSd-Bsd X 1.16.A1-Sat/Hyg **(NHxH)**	Bsd-Sat/Hyg	139/113	2/0	1.4%
**6**	LmSd-Bsd X 1.16.A1-Sat/Hyg **(NHxH)** LmSd-Bsd x LmLv39-Hyg **(NHxNH)**	Bsd-Sat/Hyg Bsd-Hyg	62/43 31	1/0 7	1.6% 23%

1. Midgut homogenates lacking bacterial/fungal overgrowth in culture.

2.NH, non-hybrid; H, hybrid

Since intraspecies F1 hybrids have so far been generated by outcrossing *L*. *major* strains of discrete geographic origin, we considered the possibility that the mating efficiency of the F1 hybrid might be improved if outcrossed with a third *L*. *major* strain unrelated to the original parents. We have previously confirmed the mating competency of a *L*. *major* strain originating in Senegal, West Africa, LmSd/BSD [16]. In the two outcross experiments involving flies co-infected with 1.16.A1 and LmSd/BSD, hybrids were recovered from 3 of 201 flies (1.5%) (Table 2, expts 5&6), all of which were PCR positive for SAT and BSD (S2C Fig). Co-infections with LmSd/BSD and LmLV39/HYG yielded a higher frequency of hybrid recovery from 7 of 31 flies (22.6%) (Table 2, expts 6, S2D Fig). Together, the backcross and outcross mating attempts indicate that while not sterile, the intraspecies F1 hybrid had reduced fertility compared to the parents used for their generation.

### Recombination in *L*. *major* backcross and outcross hybrids

**Fig 3:**
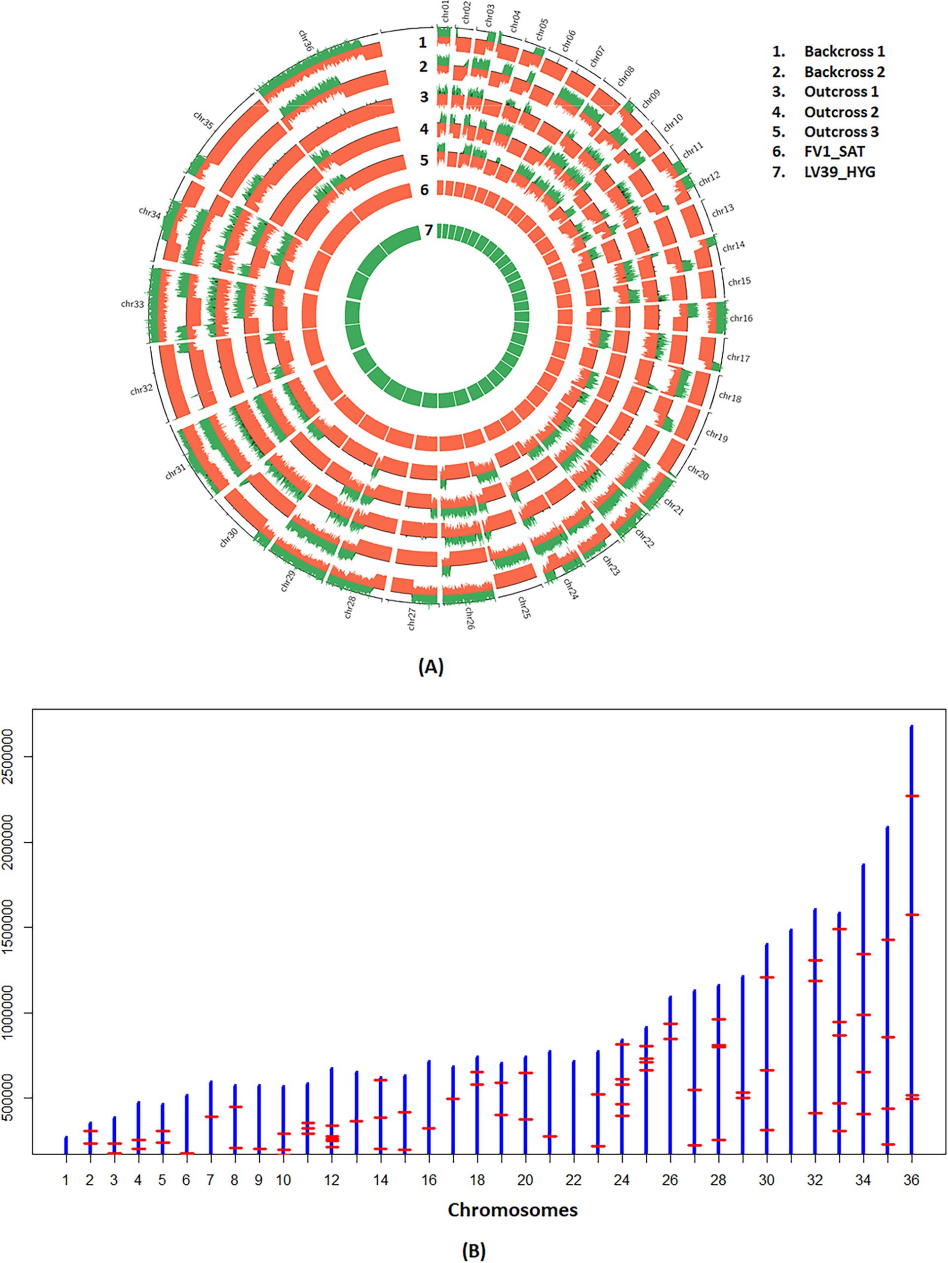
Genome-wide zygosity profiles and recombination breakpoints of the *L*. *major* backcross and outcross hybrids. **A)** The parental allele frequencies determined using AGELESS are depicted in bottle brush layout using circos plots. The height of the bars indicates contributions from parental lines shown in red for LmFV1/SAT on positive y axis and in green for LV39/HYG on negative y-axis. Boundaries between heterozygous regions where contributions from both parents are observed and the homozygous regions where contribution from only LmFV1/SAT is observed, identify the recombination breakpoints. **B)** The recombinations observed in the 5 hybrid clones generated between the F1 hybrid, 1.16.A1, and either LmFV1/BSD or LmSd/BSD are plotted by chromosome, which are represented by the blue vertical bars that are roughly scaled to the chromosome lengths. The red horizontal bars indicate the collective recombination breakpoints observed in the 5 hybrids.

The availability of 2 backcross and 3 outcross progeny allowed us for the first time to test for the presence of recombination events characteristic of meiosis in other organisms. To extract recombination patterns in outcrosses, we considered a total of 37368 markers that were common to the LmFV1/SAT and LmSd/BSD parental lines, but homozygous different from the LmLV39/HYG line, effectively treating the outcross progeny as backcrosses. The parental inheritance patterns were depicted as bottle brush plots and the recombination loci were determined visually. The schematic in S5A Fig shows the possible inheritance profiles for the backcrosses and outcrosses, assuming a maximum of 2 recombinations based on random assortment, crossovers and selection. The actual profiles of the backcrosses and outcrosses indicate that each of the expected profiles was observed (representative examples shown in S5B Fig). We incorporated the bottle brush plots into circos plots [36] depicting the allelic contributions genome wide, revealing regions of homozygosity and heterozygosity that allowed us to visualize the recombination patterns (Fig 3A). Based on these plots, we compiled the recombination loci on each chromosome to generate a physical map (S2 Table, Fig 3B). Each of the backcrosses and outcrosses had between 17 and 25 recombination events across 36 chromosomes, 21 recombinations per genome on average, or 1 recombination / 1.54 Mb. Backcrosses 1 and 2 each had 17 chromosomes with a single cross-over, and 1 or 3 chromosomes, respectively, that had two cross-overs. Outcrosses 1,2, and 3 had 17 single and 1 double, 15 single and 1 double, and 23 single and 1 double recombinations, respectively. We found a total map size of 1840 centimorgan (cM) across the 32 Mb genome which translates to 1 cM per 17,391 bp. Physical length correlated with genetic length using a linear regression model (p = 0.003). Too few backcrosses and outcrosses were available for sequencing to draw meaningful conclusions regarding recombination hotspots or coldspots.

### The mating competency of natural hybrids in *L*. *tropica*

To test the mating competency of natural hybrids in *L*. *tropica*, we introduced stable drug resistance markers into two strains, LtKub/SAT from Syria and LtRup/HYG from Afghanistan, that in our prior studies were each found to possess high levels of heterozygous SNPs (approximately 100,000 SNPs in comparison to the *L*. *tropica* L590 reference genome) in patterns consistent with these isolates being naturally occurring full genome hybrids [13]. Both strains showed robust growth in culture and in *Lu*. *longipalpis* flies (S6 Fig). Hybridization of these lines with each other or with LtMA37/NEO or LtL747/HYG, was tested in 3 independent experiments (Table 3, expts 1–3). Of the 251 flies that were co-infected with LtRup/HYG and either LtKub/SAT, LtL747/HYG, or LtMA37/NEO, no hybrids were recovered (Table 3, expt 1). By contrast, co-infection of the same population of flies with LtMA-37/NEO and LtL747/HYG, again yielded a high rate of hybrid recovery in 29 of 69 flies (42%). Ten of these hybrids were cloned and genotyped by PCR to confirm their hybrid genotypes (labeled as LtHLMB in S1 Fig). Mating attempts involving the other natural hybrid, LtKub/SAT, in two experiments yielded a total of 56 hybrid lines when paired with either LtL747/HYG or LtMA37/NEO (Table 3, expts 2&3), all of which were cloned and their hybrid genotypes confirmed (labeled respectively as LtHKLA/B hybrids in S3B Fig, and LtHKMA/B hybrids in S3A Fig). A high rate of hybrid recovery was again obtained from the same population of flies co-infected with LtMA37/NEO and LtL747/HYG (65%), 10 of which were cloned and their hybrid genotypes confirmed (labeled as LtHLMC in S1 Fig). Thus, the exceptional outcrossing efficiency in reciprocal matings of two predominantly homozygous *L*. *tropica* strains was reinforced, while the ability of two natural hybrids to mate was strain dependent, with one strain essentially sterile and the other showing good mating compatibility when tested in outcrosses with the homozygous strains.

**Table 3:** Crosses in *Lu*. *longipalpis* involving *L*. *tropica* strains.

Expt	Cross	Antibiotics	No. clean midguts	No. hybrids recovered	% hybrid recovery
**1**	LtL747-Hyg X LtMA37-Neo **(NHxNH)** ^**1**^ LtKub-Sat X LtRup-Hyg **(HxH)** LtL747-Hyg X LtRup-Neo **(NHxH)** LtRup-Hyg X LtMA37-Neo **(HxNH)**	Hyg-Neo Sat-Hyg Hyg-Neo Hyg-Neo	69 146 42 63	29 0 0 0	42% 0% 0% 0%
**2**	LtL747-Hyg X LtMA37-Neo **(NHxNH)** LtL747-Hyg X LtKub-Sat **(NHxH)** LtMA37-Neo X LtKub-Sat **(NHxH)**	Hyg-Neo Hyg-Sat Neo-Sat	52 61 69	34 20 12	65% 33% 17%
**3**	LtL747-Hyg X LtKub-Sat **(NHxH)** LtMA37-Neo X LtKub-Sat **(NHxH)**	Hyg-Sat Neo-Sat	95 102	22 2	23% 2%

1. NH, non-hybrid; H, hybrid

### Chromosome inheritance and recombination in backcross progeny in *L*. *tropica*

Eight progeny clones generated from the crosses between Lt/Kub/SAT and LtL747/HYG, and 9 clones generated between LtKub/SAT and LtMA37/NEO, designated LtHKM or LtHKL, respectively, were submitted for whole genome sequencing. This allowed us to study genome wide patterns of chromosome segregation and recombination involving a natural hybrid. The sequencing identified a high number of both heterozygous and homozygous SNPs in LtKub/SAT that were different in comparison to either Lt747/HYG or LtMA37/NEO and that were passed on to the progeny clones (Table 1D & 1E). When we enumerated the somies and the parental contributions of the chromosomes in the 567 hybrid chromosomes where each of the three parents were approximately disomic (estimated somy between 1.6 and 2.5), we found that 97% were disomic with an equal contribution from both parents, as expected under meiosis (S1 Table, S7 Fig). However, we also found a few trisomic chromosomes (14 or 3%) where an extra copy was contributed by one of the disomic parental lines, and 4 chromosomes where only the homozygous parent contributed to the hybrid (blue boxes). These 4 chromosomes were monosomic in each case, and we speculate that the chromosome contribution from the LtKub/SAT parent was lost as a consequence of a non-disjunction event during meiosis. Aneuploidy was observed in the chromosomes where one or both the parents had somies greater than 2, although of the 36 chromosomes for which the LtKub/SAT parent was trisomic (chromosomes 12 & 5) and should have had an equal opportunity to contribute a double copy, a single copy contribution was observed 92% of the time. The skewed single copy contribution of the trisomic chromosomes might have occurred by chance, or perhaps by haplotype selection during adaptation to clonal growth in culture or in the fly [32].

**Fig 4:**
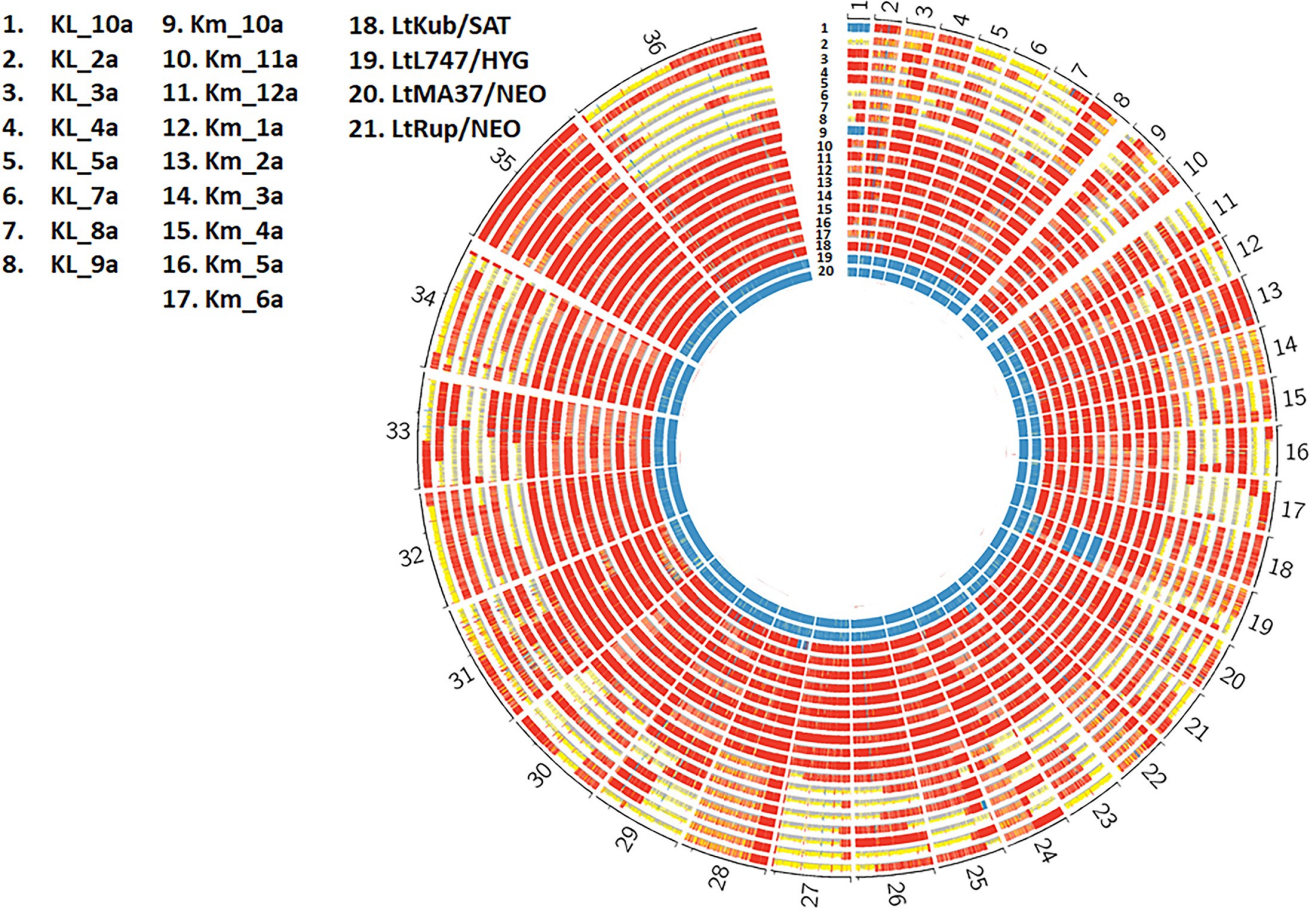
Genome-wide zygosity profiles in hybrids generated between LtKub/SAT and Lt L747/HYG (KL) or LtMA37/NEO (KM). The number of SNPs per 5kb window are depicted in red if the percentage of heterozygous SNPs was greater than 90%, in blue if the percentage of homozygous SNPs was greater than 90%, and in yellow otherwise. The patterns indicate a range of heterozygosities in the hybrids distributed in discreet blocks similar to what is commonly observed in backcrosses as a result of genetic recombination.

**Fig 5:**
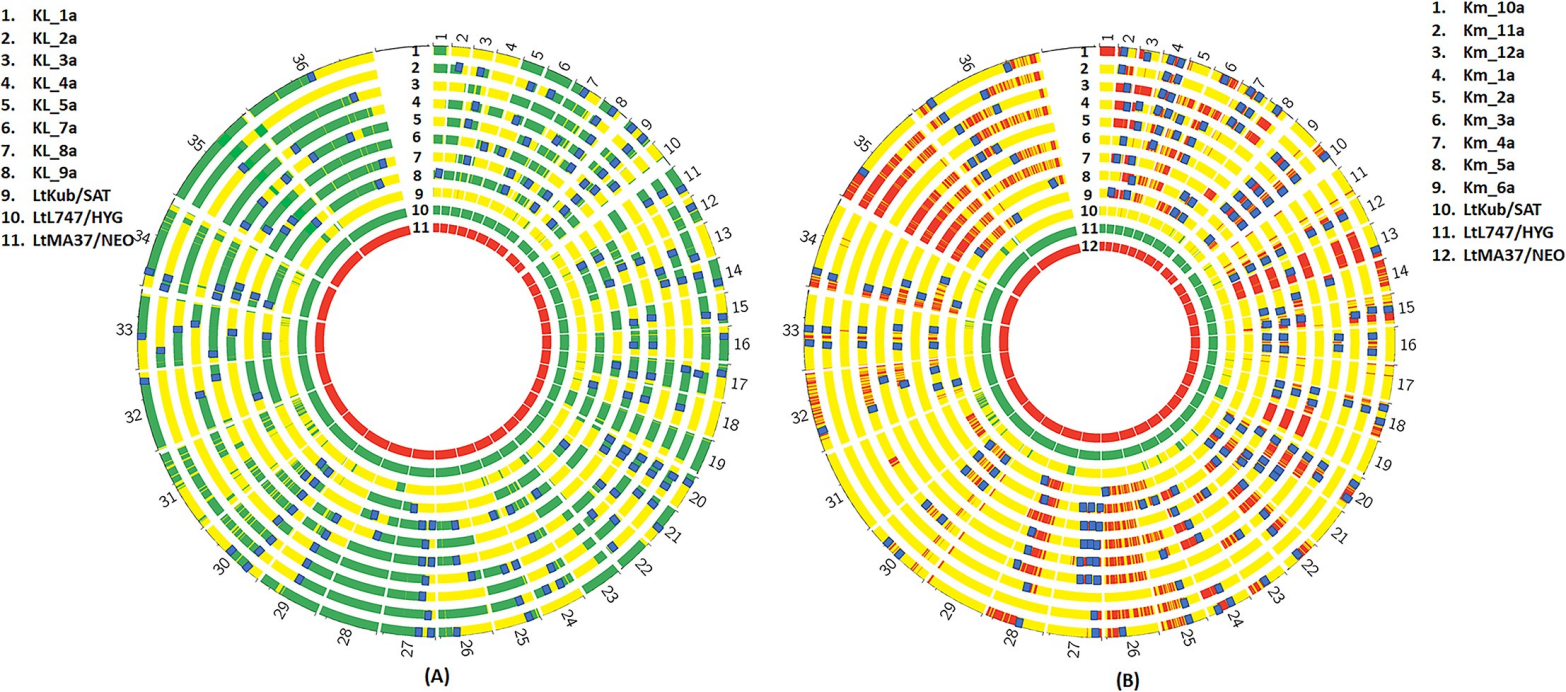
Allele composition maps reveal recombination breakpoints in *L*. *tropica* hybrids. A total of 32923 biallelic markers with homozygous differences between LtMA37/NEO and LtL747/HYG were followed. The marker position was colored as red if both alleles matched LtMA37/NEO, as green if they both matched LtL747/HYG, and as yellow if heterozygous. LtKub/SAT is 99% heterozygous for these markers, indicating that it is a hybrid between lines that share ancestry with LtL747 and LtMA37. The profiles of the outcrosses of LtKub/SAT with LtL747/HYG (left plot) or with LtMA37/NEO (right plot) indicate regions that alternate between homozygosity and heterozygosity, similar to backcrosses. The recombination break points are indicated by the blue bars.

We next constructed the zygosity profiles of the hybrids between LtKub/SAT and LtL747/HYG or LtMA-37/NEO. We identified the SNPs against the *L*. *tropica* L590 reference genome using SAMtools utility [37] and filtered out all the markers with coverage less than 10. We tagged the SNPs as homozygous if the major allele frequency was greater than 90% and as heterozygous if both the major and minor allele frequencies were between 15% and 85%. We divided the genome into blocks of 5kb and enumerated the heterozygous and homozygous SNP counts within each window. We colored the block red if the heterozygous proportion within the block was greater than 90%, blue if the homozygous proportion was greater than 90% and yellow otherwise. LtKub/SAT was mostly heterozygous while the other parental strains, LtMA37/NEO and LtL747/HYG were homozygous, as expected (Fig 4). By contrast, the outcrosses contained blocks of long runs of homozygosity, heterozygosity, or sequences that were neither homozygous nor heterozygous, similar to the patterns that are observed in the backcross progeny (see Fig 3). This suggested that the LtKub hybrid shares haplotypes with those present in LtMA37 and LtL747, and therefore might be a natural cross between strains containing genotypes similar to these strains. To test this possibility, we used a new population genetics software (https://popsicle-admixture.sourceforge.io) and performed the analysis as follows: we leveraged 159900 homozygous SNPs in LtMA37/NEO against the reference strain (TritrypDB L590 V.33) and removed 134846 homozygous SNPs that were common with LtL747/HYG. The SNPs at the remaining 23775 markers were homozygous and different between LtMA37/NEO and LtL747/HYG. We created separate Circos plots for the two groups of outcross progeny, and redrew the plots by coloring the SNPs as green if they matched Lt747/HYG, red if they matched LtMA37/NEO, and yellow if they were heterozygous (Fig 5). As expected, Lt747/HYG and LtMA37/NEO were both homozygous and contained alternate genotypes. LtKub/SAT was mostly heterozygous due to contributions from both Lt747- and LtMA37- like genotypes. The outcrosses between Lt747/HYG and LtKub/SAT contained longs runs of heterozygous and homozygous SNPs similar to backcrosses, and the homozygous regions matched Lt747/HYG. Similarly, the outcrosses between LtMA37/NEO and LtKub/SAT contained long runs of heterozygous and homozygous SNPs for which the homozygous regions matched LtMA37/NEO. These results strongly support the hypothesis that LtKub is a hybrid between strains that contained alternate genotypes matching Lt747 and LtMA37.

**Fig 6:**
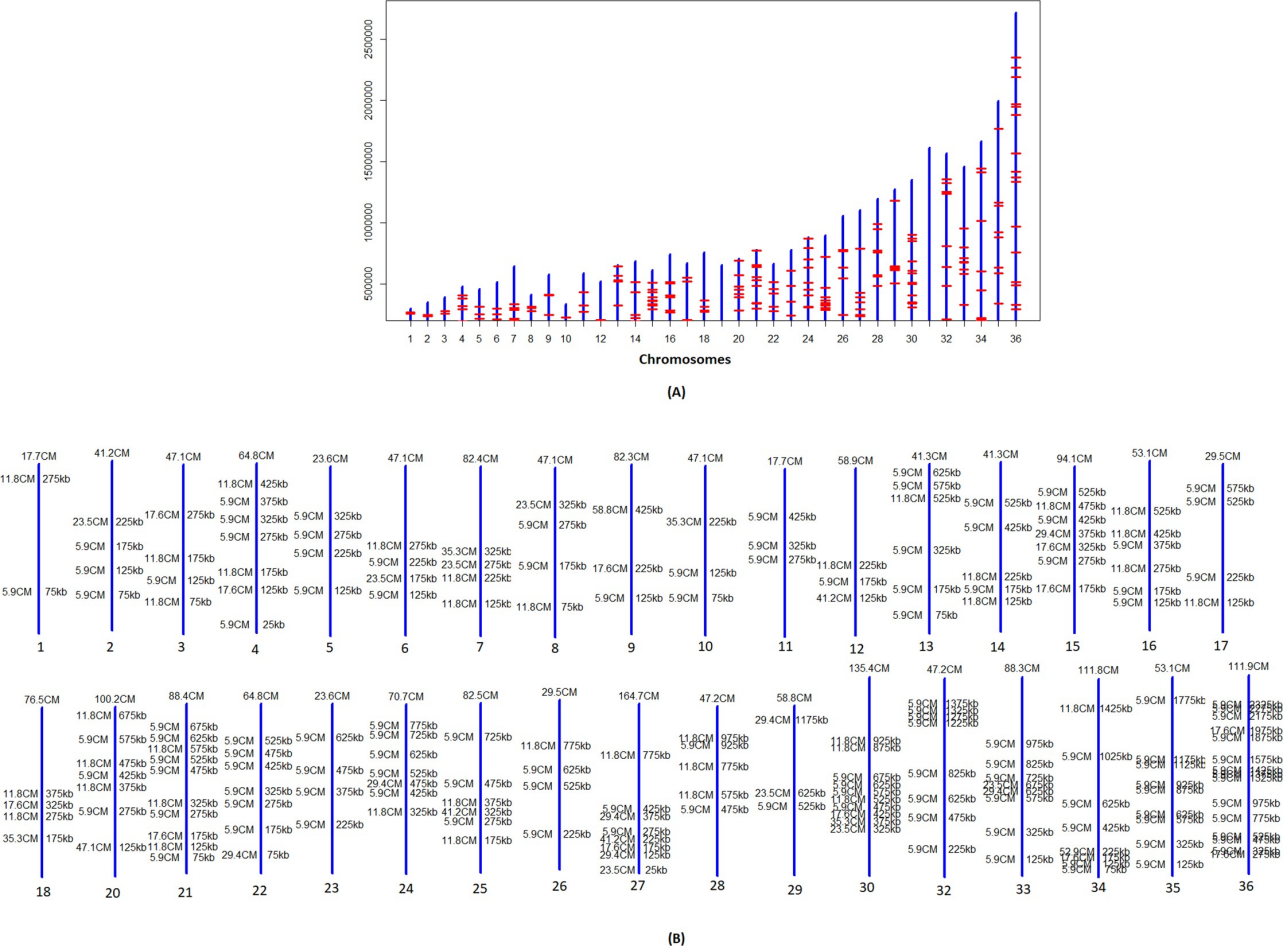
Physical and genetic maps of *L*. *tropica* hybrids. A) Physical map showing the collective recombination breakpoints across the genome observed in 17 *L*.*tropica* hybrids generated between LtKub/SAT and Lt L747/HYG or LtMA37/NEO. The break points, represented by the red horizontal bars, are plotted by chromosome which are represented by the blue vertical bars that are roughly scaled to the chromosome lengths. Although the recombinations were distributed across the genome, there were apparent hotspots, defined as more than 2 recombinations in a 50kb window (highlighted by circles), and cold spots, including whole chromosomes, where few or no recombinations were found. **B)** A genetic map was constructed based on the crossovers observed in the 17 *L*. *tropica* hybrids. Centimorgan distances were calculated in windows of 20kb across the genome by counting total recombinations across all samples and dividing by total sample size. The total centimorgan distances for each chromosome are indicated at the top of the chromosome. The chromosome lengths are not true to scale, but are arranged from small to large as shown in Fig 6B. The total genome-wide map size is 2091.4 cM with an average of 61.5 cM/chromosome.

The transitions between the long runs of heterozygous and homozygous regions are the recombination breakpoints (S2 Table), as highlighted on the Circos plots in which both single, double, and triple crossovers were observed. The hybrids between LtKub/SAT and LtL747/HYG, and between LtKub/SAT and LtMA37/NEO recorded an average of 22 recombinations each (S2 Table), which translates to 1 recombination every 1.45Mb. Double and triple crossovers were routinely observed in the hybrids between LtKub/SAT and LtMA37/NEO in comparison to the hybrids between LtKub/SAT and LtL747/HYG (S2 Table; p-value of 0.0082 using t-test). The recombinations observed across the 17 hybrids plotted by chromosome indicated that although the crossover points were distributed throughout the genome, certain hotspots (more than 2 recombinations in a 50kb window) were observed in 18 chromosomes (Fig 6A). The larger chromosomes on an average contained more recombinations in comparison to the smaller chromosomes as evaluated by linear regression (p-value of 0.009). We translated the observed recombinations into cM distances by calculating probability of finding recombinations across the genome in sliding non-overlapping blocks of 20Kb (see methods) and drew a genetic map based on the blocks that contained recombinations (Fig 6B). We found a total map size of 2091.4 cM across the 32 Mb genome, which translates to 1 cM per 15,300 bp, very close to the recombination frequency that we recorded for *L*. *major*.

### The interspecies F1 hybrids appear to be mating incompetent

Lastly, a series of backcross and outcross mating attempts involving the interspecies hybrids were undertaken in *Lu*. *longipalpis* flies. In 8 independent experiments (Table 4), the flies were co-infected with a pool of the 4 near diploid F1 hybrids (H2, H4, H7, H6), or with each of the hybrids individually, and paired with either LmFV1/BSD or LmSd/BSD. The midgut promastigotes were selected for growth in either SAT + BSD or HYG + BSD. From a total of 420 co-infected flies that could be evaluated for hybrid recovery, no backcross or outcross progeny were obtained (Table 4). Thus, the interspecies hybrids appear to be sterile under the mating conditions employed. By comparison, when the flies were co-infected with the parental lines LiL/HYG and LmSd/BSD, a total of 10 of 140 flies (7.1%) yielded hybrids, with recovery rates in the different experiment ranging from 3% to 23%. PCR tests confirmed that the progeny clones generated from these 10 lines contained both parental selectable markers (hybrids labeled as LimHLS, S4 Fig).

**Table 4:** Backcross/Outcross matings in *Lu*. *longipalpis* involving interspecies F1 hybrids.

Expt	Cross	Antibiotics	No. clean midguts	No. hybrids recovered	% hybrid recovery
**1**	LmFV1-Bsd X LimH2-Sat/Hyg **(NHxH)**^**1**^	Bsd-Sat/Hyg	82/68	0/0	0%
**2**	LmFV1-Bsd X All LimH-Sat/Hyg **(NHxH)**	Bsd-Sat/Hyg	54/33	0/0	0%
**3**	LmFV1-Bsd X LimH4-Sat/Hyg **(NHxH)**	Bsd-Sat/Hyg	53/50	0/0	0%
	LmFV1-Bsd X LimH7-Sat/Hyg **(NHxH)**	Bsd-Sat/Hyg	46/38	0/0	0%
**4**	LmSd-Bsd X LimH4-Sat/Hyg **(NHxH)**	Bsd-Sat/Hyg	67/69	0/0	0%
**5**	LmSd-Bsd X LimH6-Sat/Hyg **(NHxH)**	Bsd-Sat/Hyg	144/148	0/0	0%
**6**	LmSdBsd X LimH2-Sat/Hyg **(NHxH)**	Bsd-Sat/Hyg	120/126	0/0	0%
**7**	LmSd-Bsd X LimH2-Sat/Hyg **(NHxH)**	Bsd-Sat/Hyg	23/19	0/0	0%
	LiL-Hyg x LmSd-Bsd **(NHxNH)**	Hyg-Bsd	67	2	3%
**8**	LmSd-Bsd X LimH10-Sat/Hyg **(NHxH)**	Bsd-Sat/Hyg	66/75	0/0	0%
	LiL- Hyg x LmSd-Bsd **(NHxNH)**	Hyg-Bsd	35	8	23%

1. NH, non-hybrid; H, hybrid

## Discussion

We present here the first comprehensive analysis of experimental intra- and interspecies hybrids in *Leishmania* by analyzing high-resolution whole genome sequencing data. We determined the chromosomal somy and studied the parental inheritance of 44 hybrids generated within and between different Old World species of *Leishmania*, including *L*. *major*, *L*. *infantum*, and *L*. *tropica*, and compared them against the inheritance patterns expected under meiosis. The somies and parental chromosomal contributions matched the expected inheritance patterns 97%-99% of the time, supporting a predominant meiotic-like process in *Leishmania*, which we believe is the most parsimonious interpretation of the genome-wide inheritance patterns presented in this report. The hybrids appeared equivalent to F1 progeny, heterozygous throughout most of the genome for the homozygous alleles that were different between the parents. The majority of the hybrid clones that we have generated and analyzed in this report were near diploid, showing balanced segregation of the chromosomes, the majority of which were disomic in the parents. Trisomic chromosomes in the parents were passed on to the progeny in single or double copy, never in their original trisomic state, and in frequencies expected by Mendelian segregation. Tetrasomic chromosomes were passed on in double copy and not in quadruple copy. Such predictable, balanced allotments of parental chromosomes during hybridization seem highly unlikely to have arisen by a random, parasexual process. While it is true that the meiotic intermediates in aneuploid lines of *Leishmania* would not be strictly haploid, aneuploidy is not incompatible with meiosis. Meiotic chromosome segregation is well described in triploid strains of *S*. *cerevisiae* which accurately produce viable tetrads containing 2 spores with 2 copies and 2 spores with 1 copy of each homolog [38]. Furthermore, all 3 copies of a trisomic chromosome in a close to diploid strain of *S*. *cerevisiae* were shown to undergo recombination in a single meiosis [39]. In *Drosophila*, although the fertility of triploid females is reduced, viable offspring between diploids and triploids can be readily obtained [40]. Finally, it is worth noting that some women with non-mosaic trisomy 21 are fertile and pass on the extra chromosome to a high proportion of their offspring [41].

Polyploid, predominantly triploid hybrids, have been routinely recovered from experimental matings in *Leishmania* [15–17], and are observed across organisms capable of sexual reproduction, such as amphibians [42], plants [43], and fruit flies [40]. The genomic analysis of 4 interspecies hybrids with close to 3n DNA content showed parental contributions consistent with syngamy between a parental ‘2n’ cell that failed to undergo meiosis and a ‘1n’ cell from the other parent, similar to what has been suggested for *T*. *brucei* [44] for which triploid progeny are also common. Their inheritance profiles again argue against a parasexual process involving random chromosome loss from a tetraploid intermediate that would seem highly unlikely to produce progeny for which almost all of the chromosomes that were disomic in each parent were allotted one extra copy, with the extra copies within each clone always contributed by the same parent.

Our recovery of a few hybrids with close to 4n DNA content, one of which was analyzed here, is interesting because they suggest that fusion of diploid cells producing a tetraploid hybrid can indeed occur in *Leishmania*. The progeny clone analyzed remained close to tetrasomic for the majority of chromosomes. While it might be argued that this hybrid represents a parasexual intermediate that has not yet experienced chromosome loss, a stronger case might be made for a tetraploid meiotic cycle, originally described for *Saccharomyces* [45], for which diploid cells of two different mating types fuse and then undergo meiosis followed by fusion of haploid nuclei to produce diploid progeny. The triploid and tetraploid hybrids in *Leishmania* might be the result of one or both of the parental nuclei failing to undergo meiosis following fusion of the diploid cells. While the tetraploid meiotic cycle does not involve the generation of gametes, it is still referred to in the context of a sexual reproductive cycle in yeast [46]. Sexual reproduction can be generalized to mean all forms of meiotic reproduction in protists, which frequently retain the ability to reproduce asexually via mitosis.

The instances of unbalanced chromosome inheritance that might support a parasexual process in *Leishmania* were the exceptions, not the rule. Specifically, they were manifested as a gain of somy in comparison to either parent, observed approximately 2% of the time, and uniparental inheritance, observed approximately 1% of the time, that we interpret as LOH subsequent to the original hybridization event. Non-disjunction of one parental chromosome at meiosis or during subsequent mitotic generations in the fly or in culture seem more likely to explain the instances of trisomy than a parasexual process, which would be expected to lead to high chromosome dosage genome-wide. An error-prone meiosis would not be unique to *Leishmania* genome biology. For example, the fungus *Candida lusitaniae* has a well defined sexual cycle during which aneuploid hybrids frequently arise [47]. Alterations in chromosome copy number are also known to arise during in vitro cultivation of *Leishmania* promastigotes [48, 49], so may have occurred during mitotic division in the fly or during the in vitro selection and cloning procedures. The emergence of new aneuploidies during clonal growth may reflect a process of haplotype selection [32], driven by adaptation to the growth conditions in the fly or in culture. Mosaic aneuploidy can lead to LOH during mitotic divisions [26], or a partially homozygous state should crossing over events also occur.

Meiosis also involves frequent reciprocal crossover events between homologous chromosomes, and is considered an essential process for the two homologues to establish the physical connections needed to orient properly during the first meiotic division [50]. Whole genome sequencing of the progeny generated from crosses involving an experimental F1 hybrid of *L*. *major*, or a natural hybrid of *L*. *tropica*, bearing in each case a high number and genome wide distribution of heterozygous alleles, provides the first clear evidence for recombination events in *Leishmania*. The very high levels of interhomolog recombination observed have not been reported in parasexual organisms, so far as we are aware. For example, while recombination between homologous chromosomes was observed during the parasexual cycle in *C*. *albicans*, only 3 of 13 progeny strains showed evidence of mitotic crossing-over [24]. By comparison, of the backcross and outcross hybrids that were generated in the present study and for which homologous recombination could be properly assessed, all 22 revealed genome wide crossover events. This analysis allowed us to calculate the best estimate to date of recombination frequencies, and to construct the first physical maps of recombination break points in *Leishmania*. The backcross and outcross progeny generated from the *L*. *major* hybrid revealed discrete blocks of homozygous and heterozygous SNPs throughout the genome that would be expected from meiotic recombination and random segregation of the homologous chromosomes in the hybrid parent. The haplotypes of the heterozygous alleles in LtKub/SAT implied a relationship to both the LtMA37/NEO and LtL747/HYG parents with which it was crossed, reflected in the progeny genotypes showing discrete blocks of homozygosity and heterozygosity that would be expected from backcross matings. The recombination frequencies for the *L*. *major* and *L*. *tropica* backcrosses were similar and averaged 1 cross-over per 1.5Mb. The only other estimate of recombination frequency in *Leishmania*, 1 cross-over event per 2.3Mb per generation, was calculated from the levels of recombination observed in natural sand fly and human isolates of *L*. *infantum* obtained from a transmission focus in Turkey [14]. For our experimental crosses, the average recombination unit for *L*. *major* and *L*. *tropica* was 1 cM per 17,391 bp and 15,300 bp, respectively. These metrics compare well with that calculated for *Trypanosoma brucei* [23], which has a map length of 733 cM across the 17.89 Mb genome, or 1 cM per 24,406 bp.

Our studies of the mating compatibilities of experimental F1 hybrids suggested that the intraspecies *L*. *major* hybrid had reduced mating competency in comparison to the parents used for its generation, while each of the interspecies *L*. *major* x *L*. *infantum* hybrids were mating incompetent, at least under the mating conditions employed. Reproductive barriers arising as a result of hybrid sterility are well described across phylogeny, and are the most common form of postzygotic reproductive isolation in plants [51–53]. In *Saccharomyces* yeast, F1 hybrids can reproduce normally by asexual budding, however, the spores they make are inviable and sexually sterile [51]. A variety of underlying mechanisms have been described to account for yeast hybrid sterility, including chromosomal rearrangement [54], aneuploidy [55], and sequence divergence acted on by mismatch repair [56], where pairing, recombination and segregation fail because the parental homologous chromosomes are not sufficiently alike. It is interesting that for the cross-species hybrids, which are so far ‘sterile', the sequence divergence between the hybridizing genomes of the parents responsible for their generation is approximately 10-fold greater than the sequence divergence between the parental genomes of the intraspecies hybrids, which remain mating competent, though at reduced efficiency. It is notable, however, that the two presumed natural *L*. *tropica* hybrids, LtKub/SAT and LtRup/HYG, showed vastly different mating potentials despite their similar levels of heterozygosity. In *T*. *brucei*, the success of experimental backcrosses was also variable, suggesting a range of mating compatibilities among F1 progeny [57]. Our experience with LtRup/HYG aside, the successful crosses involving 3 other *L*. *tropica* strains has provided the first direct demonstration of genetic exchange between members of this species, and strongly supports the conclusion that the hybrid genotypes found especially prevalent in natural populations of *L*. *tropica* are in fact products of meiotic outcrossing events [13, 34, 35].

In summary, the whole genome sequencing analysis of experimental hybrids generated within and between different Old World species of *Leishmania*, provides the clearest evidence to date that the system of genetic exchange is Mendelian and involves meiosis-like sexual recombination. These findings, along with the demonstration that it is possible to backcross *Leishmania* in the laboratory, show that the forward genetic tools are available for linkage studies and positional cloning of important genes.

## Materials and methods

### Parasites

A summary of the parental lines that were used to generate the experimental hybrids analyzed in this report, including their heterozygous drug resistance loci, is provided in S3 Table. The following lines of *L*. *major* were used: LmFV1/BSD (MHOM/IL/80/Friedlin) was originally isolated from a patient with cutaneous leishmaniasis (CL) acquired in the Jordan Valley, and is heterozygous for an allelic replacement of *LPG5B* on chromosome 18 by a blasticidin S-resistance (BSD) marker [58]; LmSd/BSD was previously described [16] and is derived from a strain isolated from a patient with CL acquired in Senegal (MHOM/SN/74/SD) [59] and contains the same allelic replacement of *LPG5B*; LmLV39/HYG [15] is derived from a strain originally isolated from a reservoir rodent host in southern Russia (MRHO/SU/59/P-strain) [60] and is heterozygous for an allelic replacement of on chromosome 24 by a hygromycin B–resistance cassette [58]; Lm1.16.A1 is a previously described intraspecies hybrid [15] generated by a cross between LmFV1/SAT and LmLV39/HYG. LiL/HYG [17] is derived from *L*. *infantum* (MHOM/ES/92/LLM-320; isoenzyme typed MON-1) [61], isolated from a human case of visceral leishmaniasis (VL) in Spain, and is heterozygous for a hygromycin B resistance cassette integrated into the 18S rDNA locus. Hybrids LimH2, 4, 6, 7 and 10 were previously generated [17] interspecies hybrids from crosses between LiL/HYG and LmFV1/SAT. The following *L*. *tropica* lines were generated from strains described previously [13]: *L*. *tropica* LtMA37 (MHOM/JO/94/MA37) was originally isolated from a patient with CL in Jordan and is heterozygous for either a G418 (LtMA37 /NEO) or a hygromycin B (LtMA-37/HYG) resistance cassette integrated into the 18S rDNA locus on chromosome 27 using the constitutive expression vectors pLEXY neo2.1 or pLEXY hyg2.1, respectively (Jena Bioscience EGE 273 and EGE-272); LtL747/HYG (MHOM/IL/02/LRC-L747) was originally isolated from a patient with CL in Israel and is heterozygous for a hygromycin B resistance cassette, using the pLEXY hyg2.1 vector; LtKub/SAT is derived from a strain isolated from a patient with CL in Syria (MHOM/SY/?/Kub) and is heterozygous for a Nourseothricin resistance cassette (SAT) also integrated into the 18S rDNA locus on chromosome 27 using the pLEXY sat2.1 vector (Jena Bioscience EGE-274); LtRup/HYG and LtRup/NEO are derived from a strain originally isolated from a patient with CL in Afghanistan (MHOM/AF/87/RP) and are heterozygous for either a hygromycin B or G418 resistance cassette using the pLEXY hyg2.1 or pLEXY neo2.1 vectors, respectively. All lines were grown at 26^o^ C in medium M199 as described previously [16] with or without 25 μg/ml hygromycin B (HYG), 100 μg/ml nourseothricin (SAT), 50 μg/ml G-418 (NEO), 25 μg/ml blasticidin S or a combination of these drugs as necessary. The hybrid clones underwent approximately 25–28 generations in vitro prior to sequencing.

### Sand fly infections and hybrids recovery

*Lutzomia longipalpis* and *Phlebotomus duboscqi* sand flies, collected from field specimens in Brazil and Mali, respectively, were infected by artificial feeding through a chick skin membrane on heparinized mouse blood as previously described [16]. The mouse blood was obtained in strict accordance with the recommendations in the Guide for the Care and Use of Laboratory Animals at the NIH. The protocols were approved by the Animal Care and Use Committee of the NIAID (protocol LPD 68E).

For the generation of hybrids, the blood was seeded with an equal mixture of the two parental lines containing independent drug resistant markers at 4–8 x10^6^ logarithmic phase promastigotes /ml of blood. Midguts were dissected after 7–11 days, and hybrid parasites were recovered by double drug selection as described previously [16]. For the generation of backcross (BC) or outcross hybrids (OC) using an F1 hybrid as one of the parents, flies were coinfected with the F1 hybrid clone (already double drug resistant) and one of the parental lines or an unrelated parent, each harboring a third antibiotic resistance marker. Flies were dissected and the guts were homogenized in M199 and plated in 96-well culture plates without selection. One day following plating, each well was subdivided into two wells containing either one of the drugs for which the F1 parent is resistant, and the third drug for which the other parental line is resistant (e.g. HYG-BSD and SAT-BSD). Notably, some of the BC or OC lines were resistant to all three drugs. Double/triple drug resistant BC and OC lines were cloned as described previously [15].

### Ploidy, DNA extractions and hybrids genotyping by PCR

Total DNA content and inferred ploidy was determined on the different parental lines and hybrid progeny clones (see previous section) using flow cytometry following staining of permeabilized RNAse-treated cells with propidium iodide, as previously described [16]. Genomic DNA was extracted from the parental lines and hybrid clones using DNeasy blood & Tissue kit (Qiagen #69506) according to the manufacturer instructions. PCR amplification of the drug selectable markers was carried out as previously described [17] using high fidelity PCR mix MyFi 2X (Bioline #25050). PCR products were cleaned using ExoSAP-IT, PCR cleanup reagent (ThermoFisher #78200). Amplified products were verified by electrophoresis on a 1.5% (wt/vol) agarose gel and visualized by ethidium bromide staining.

### Whole genome DNA sequencing and read alignments

DNAs extracted from most of the lines (see previous section) were submitted to whole genome sequencing in Rocky Mountain Laboratory’s Research Technologies Section-Genomics Unit, Division of Intramural Research, NIAID, NIH. DNAs were sheared using the Covaris LE220 for a 200bp insert target size and the fragmented genomic DNA was purified using AMPure XP beads. Libraries were generated with the ThruPLEX DNA-Seq kit (Rubicon Genomics) and quantified using the Kapa SYBR FAST Universal qPCR kit for Illumina sequencing (Kapa Biosystems, Boston, MA). The libraries were diluted to 2 nM stocks and pooled equally. The 2 nM pooled stock was prepared for sequencing by denaturing and diluting to 11 pM for clustering to a Rapid flow cell. On-board cluster generation and paired-end sequencing was completed on the HiSeq 2500 (Illumina, Inc, San Diego, CA) using a Rapid Paired End cluster kit and 200 cycle sequencing kit. The cluster density averaged 990 k/mm2 per lane resulting in 163 M reads passing filter per lane with 95% > Q30. The samples were sequenced to target 60x coverage, and achieved median coverages across the genome per sample of between 19 and 38x coverage.

Similar methods were used for DNA extracted from the 17 intraspecies *L*. *tropica* progeny and parental clones which were sequenced in DNA pipelines at Wellcome Sanger Institute (WSI). Differences were that DNA was sheared to a 400bp insert target, and libraries were generated with the NEBNext DNA Library Prep kit (New England BioLabs). These libraries were diluted to 4 nM stocks, and the 4nM pooled stock was diluting to 8 pM for clustering. The cluster density averaged 550 k/mm2 per lane resulting in at least 100 M reads passing filter per lane with 95% > Q30. The raw data was deposited in the European Nucleotide Archive (ENA) in study ERP111791. These samples were sequenced to target 30x coverage, and after removal of duplicates, achieved median coverages across the genome per sample of between 19 and 38x coverage.

All samples were subjected to whole genome sequencing using the Illumina Hi-Seq 2500 platform. The 100bp paired end reads were aligned to the closest available reference genome (Tritrypdb, *L*. *major* FV1 Version 6, *L*. *infantum* JPCM5 Version 6, *L*. *tropica* L590 V. 33) using novoalign software, and using the parameters -F (format) ILMFQ for Illumina 1.3 pipeline, -H enables hard clipping that removes low quality bases from the 5’end, -g 40 for gap opening penalty, -x 6 for gap extend penalty, -R 5 for difference in score between the best alignment and the second best alignment, -r none ignores the reads with similar scores to multiple locations in the genome, -e 1000 for maximum number of alignments for a single read.

### Determination of somies, alleles, and parental chromosomal inheritance

Somies were determined using AGELESS software (http://ageless.sourceforge.net/) by dividing the chromosomes into blocks of 5kb and average coverage within each block was calculated. Punctate regions with coverage greater than twice the average coverage for each chromosome or less than half the average coverage for each chromosome were ignored for somy determination. These regions are associated with repeats, gene families and other genomic noise that alter the somy values. The blocks with zero coverage were ignored from further analyses as they indicate noisy/repeat regions filtered in the previous step. The average of block coverages was scaled to the ploidy of the organism to determine the somies.

The alleles in each sample were also determined using AGELESS software. The nucleotide compositions at loci with coverage greater than or equal to 5 were considered and the rest were reported as missing data. The loci that met the minimum coverage criteria were queried for allele frequencies. Only those alleles with frequencies between 0.15 and 1 were reported; alleles with frequencies less than 0.15 were ignored as noise. The alleles with frequencies greater than 0.90 were tagged homozygous and those with frequencies between 0.15 and 0.85 were tagged heterozygous.

Parental chromosomal inheritance patterns determined using AGELESS indicate how many chromosomes were inherited from each parent. The homozygous SNP differences between the parents were used as markers and frequencies of alleles inherited from each parent were calculated in the hybrids. These parental allele frequencies when multiplied by somy reveal the number of chromosomes inherited from each parent.

### Determination of conserved regions between different species

The analysis of the interspecies hybrids was limited to genomic regions that were conserved between the species. The conserved regions were determined by a read mapping-based strategy, and represented approximately 92% of the genomes of each species. The reads from one parental line were aligned to the reference genomes of both the species, *L*. *major* FV1 Version 6 and *L*. *infantum* JPCM5 Version 6. The reads that aligned to both the genomes were retained, and independently, the reads from the other parental line were also aligned to the genomes of both species. The conserved regions from this alignment were compared against the conserved regions from alignments to the other parent to retain the regions common to both the alignments.

### Construction of genetic map

We constructed the genetic map based on the recombinations observed in *L*. *tropica* hybrids. We divided the genome into segments of 20kb and counted the number of recombinations in each segment. We calculated the probability of observing a recombination in each block by dividing the recombination counts with total hybrids employed in our analysis. Since a centimorgan (cM) is 1% probability of finding a recombination, the calculated probabilities were converted to cM distances by multiplying by 100.

## Supporting information

S1 Table Somy values of parents and hybrids. (XLSX)

S2 Table Crossover events in LtKub-SAT x LtL747-HYG/MA37-NEO hybrids. (DOCX)

S3 Table Parental lines used for generation of experimental hybrids. (DOCX)

S1 Fig PCR for parental drug resistance markers in *L*. *tropica* hybrids. PCR was done on DNA extracted from the parental lines LtMA37/NEO and LtL747/HYG, and from 29 hybrids (LtHLM) generated by crossing these two lines in three different experiments (A, B and C). Blank is PCR mix with no template.
(TIF)

S2 Fig PCR for parental selectable drug markers in intraspecies *L*. *major* hybrids. A) PCR for SAT, HYG and BSD resistance genes on the parental lines LmFv1/Sat, LmLV39/HYG, 1.16A1 –hybrid generated by these two lines, LmFv1/BSD and two backcross lines, BXLmA and BXLmB generated by crossing LmFv1/BSD and 1.16.A1. B) PCR for HYG and SAT on the parental lines LmFv1/BSD and LmLV39/HYG, and 10 hybrids generated by crossing these two lines (HFL) in two different experiments (A and B). C) PCR for SAT, HYG and BSD in 3 outcross lines generated in two experiments (LmOXA and LmOXB) in which 1.61.A1 hybrid was crossed with LmSd/BSD. D) PCR for HYG and BSD in 7 hybrids generated by crossing LmSd/BSD and LmLV39/HYG. Blank is PCR mix with no template.
(PNG)

S3 Fig PCR for parental selectable drug markers in *L*. *tropica* hybrids. A) PCR on DNA extracted from the parental lines LtMA37/NEO and LtKub/SAT, and 13 hybrid clones (LtHKM), or the parental lines LtL747/HYG and LtKub/SAT, and 42 hybrid clones (LtHKL), generated in two experiments (A & B). Hybrids were recovered on selection medium containing SAT and NEO or SAT and HYG. Blank is PCR mix with no template.
(PNG)

S4 Fig PCR for parental selectable drug markers in interspecies *L*. *major* x *L*. *infantum* hybrids. PCR for HYG and BSD on DNA extracted from the parental lines LmSd/BSD and LiL/HYG, and 10 hybrids generated by crossing these two lines. Blank is PCR mix with no template.
(TIF)

S5 Fig Expected and observed patterns of chromosome assortment and recombination in backcrosses involving an *L*. *major* F1 hybrid. **A)** Schematic showing the possible recombinations assuming not more than two crossovers per chromosome. **B)** Bottle brush plots of representative chromosomes showing the possible inheritance patterns in the 5 hybrid clones generated between the F1 hybrid, 1.16.A1, and either LmFV1/BSD (BC1-2) or LmSd/BSD (OC1-3). SNPs inheritance from LmFV1 or LmSd are shown on the positive x-axis in red, and SNPs inheritance from LmLV39 are shown on the negative x-axis in green. The vertical distance corresponds to the inferred allelic depth, normalized across the entire genome, which was assigned an average somy of 2.
(PDF)

S6 Fig Growth and development of *L*. *tropica* strains in *Lu*. *longipalpis* sand flies. Flies were membrane fed on mouse blood containing with 4x10^6^ / ml of either LtRupert or LtKubba log phase promastigotes. Flies were dissected days 2 or 8 post-infection and homogenized midguts scored under a hemocytometer for the number of each of 4 different developmental stages of promastigotes. Values shown are individual flies with geometric means +/- 1 s.d., 10 flies / group at each time point.
(TIFF)

S7 Fig Somies and parental contributions in the progeny generated between LtKub/SAT and Lt L747/HYG (KL) or LtMA37/NEO (KM). The read alignments to the genome were translated into somies and depicted as a heatmap, rounded off to the nearest 0.25 value. Overlaid are the parental inheritance values in the format LtKub-SAT/LtL747-HYG or LtKub-SAT/LtMA37-NEO, rounded off to the nearest 0.1 value. Monosomic chromosomes showing a single parent contribution are indicated by blue boxes.
(TIF)
