# Supplementary material for: Whole genome sequencing of experimental hybrids supports meiosis-like sexual recombination in Leishmania
Source: PLoS Genet. 2019 May 15;15(5):e1008042. doi: 10.1371/journal.pgen.1008042 (PMC6519804; doi:10.1371/journal.pgen.1008042)
Supplement: S2 Table — (DOCX) [file pgen.1008042.s002.docx]

| **Table S2. Crossover events in LtKub-SAT x LtL747-HYG/MA37-NEO hybrids** | | | | | |
| --- | --- | --- | --- | --- | --- |
| **Progeny clone** | **Total**  **recombinations** | | **Single crossovers** | **Double crossovers** | **Triple crossovers** |
| KL_10a | 21 |  | 15 | 3 | 0 |
| KL_2a | 24 |  | 17 | 2 | 1 |
| KL_3a | 18 |  | 16 | 1 | 0 |
| KL_4a | 23 |  | 19 | 2 | 0 |
| KL_5a | 30 |  | 14 | 5 | 2 |
| KL_7a | 20 |  | 14 | 3 | 0 |
| KL_8a | 25 |  | 19 | 3 | 0 |
| KL_9a | 17 |  | 15 | 1 | 0 |
| Average | 22.2 |  | 16.1 | 2.5 | 0.3 |
|  |  |  |  |  |  |
| KM_10a | 32 |  | 16 | 8 |  |
| KM_11a | 21 |  | 9 | 6 |  |
| KM_12a | 17 |  | 9 | 4 |  |
| KM_1a | 22 |  | 13 | 3 | 1 |
| KM_2a | 21 |  | 12 | 3 | 1 |
| KM_3a | 21 |  | 12 | 3 | 1 |
| KM_4a | 23 |  | 6 | 7 | 1 |
| KM_5a | 27 |  | 7 | 7 | 2 |
| KM_6a | 15 |  | 7 | 4 |  |
| Average | 22.1 |  | 10.1 | 5 | 1.2 |

KL clones: LtKub-SAT x LtL747-HYG**;** KM clones: LtKub-SAT x LtMA37-NEO
