# Supplementary material for: Whole genome sequencing of experimental hybrids supports meiosis-like sexual recombination in Leishmania
Source: PLoS Genet. 2019 May 15;15(5):e1008042. doi: 10.1371/journal.pgen.1008042 (PMC6519804; doi:10.1371/journal.pgen.1008042)
Supplement: S3 Table — (DOCX) [file pgen.1008042.s003.docx]

| **Table S3. Parental lines used for generation of experimental hybrids** | | | |
| --- | --- | --- | --- |
| **WHO strain designation** | **Line designation** | **Heterozygous drug resistance loci** | **Reference** |
| MHOM/IL/80/Friedlin | LmFV1/BSD  LmFV1/SAT | LPG5B; chromosome 18  *SSU;* chromosome 27 | 15; 58 |
| MRHO/SU/59/P-strain | LmLV39/HYG | *LPG5A;* chromosome 24 | 15; 60 |
| MHOM/SN/74/SD | LmSd/BSD | LPG5B; chromosome 18 | 16; 59 |
| MHOM/ES/92/LLM-320 | LiL/HYG | *SSU;* chromosome 27 | 17; 61 |
| MHOM/JO/94/MA37 | LtMA37 /NEO  LtMA37/HYG | *SSU;* chromosome 27 | 13 |
| MHOM/IL/02/LRC-L747 | LtL747/HYG | *SSU;* chromosome 27 | 13 |
| MHOM/SY/?/Kub | LtKub/SAT | *SSU;* chromosome 27 | 13 |
| MHOM/AF/87/RP | LtRup/HYG  LtRup/NEO | *SSU;* chromosome 27  *SSU;* chromosome 27 | 13 |
