# Supplementary figures and images for: Whole genome sequencing of experimental hybrids supports meiosis-like sexual recombination in Leishmania
Source: PLoS Genet. 2019 May 15;15(5):e1008042. doi: 10.1371/journal.pgen.1008042 (PMC6519804; doi:10.1371/journal.pgen.1008042)

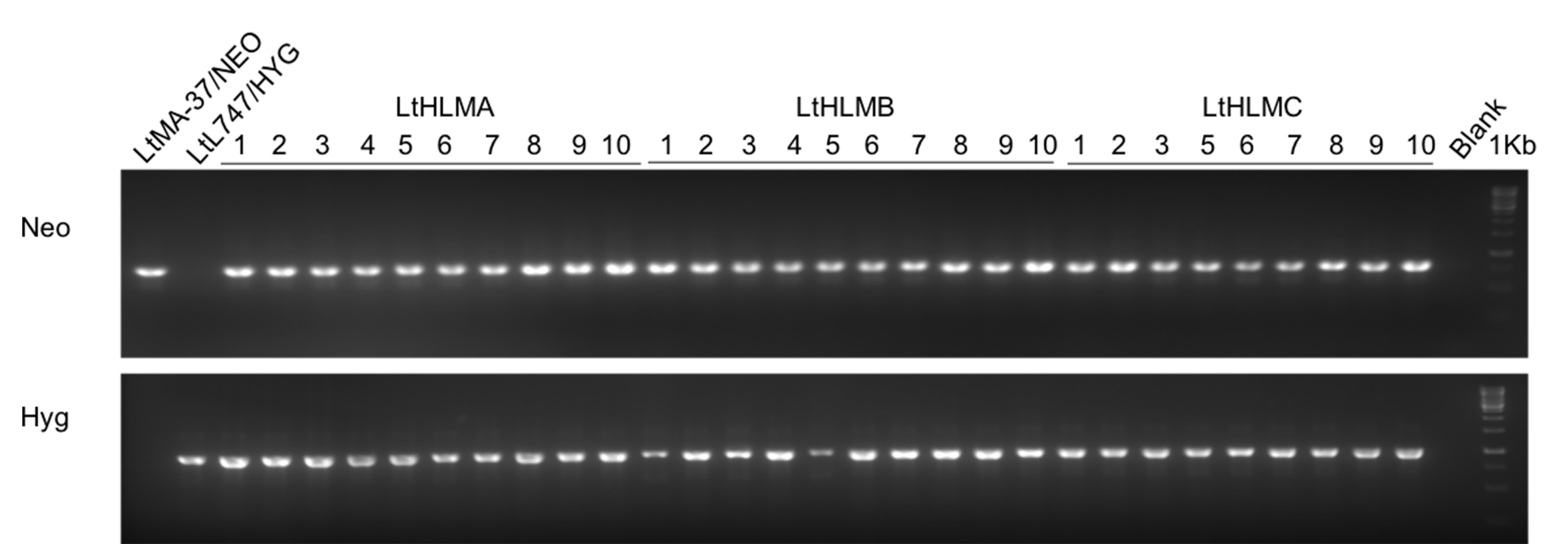

Supplement: S1 Fig — PCR was done on DNA extracted from the parental lines LtMA37/NEO and LtL747/HYG, and from 29 hybrids (LtHLM) generated by crossing these two lines in three different experiments (A, B and C). Blank is PCR mix with no template. (TIF) [file pgen.1008042.s004.tif]

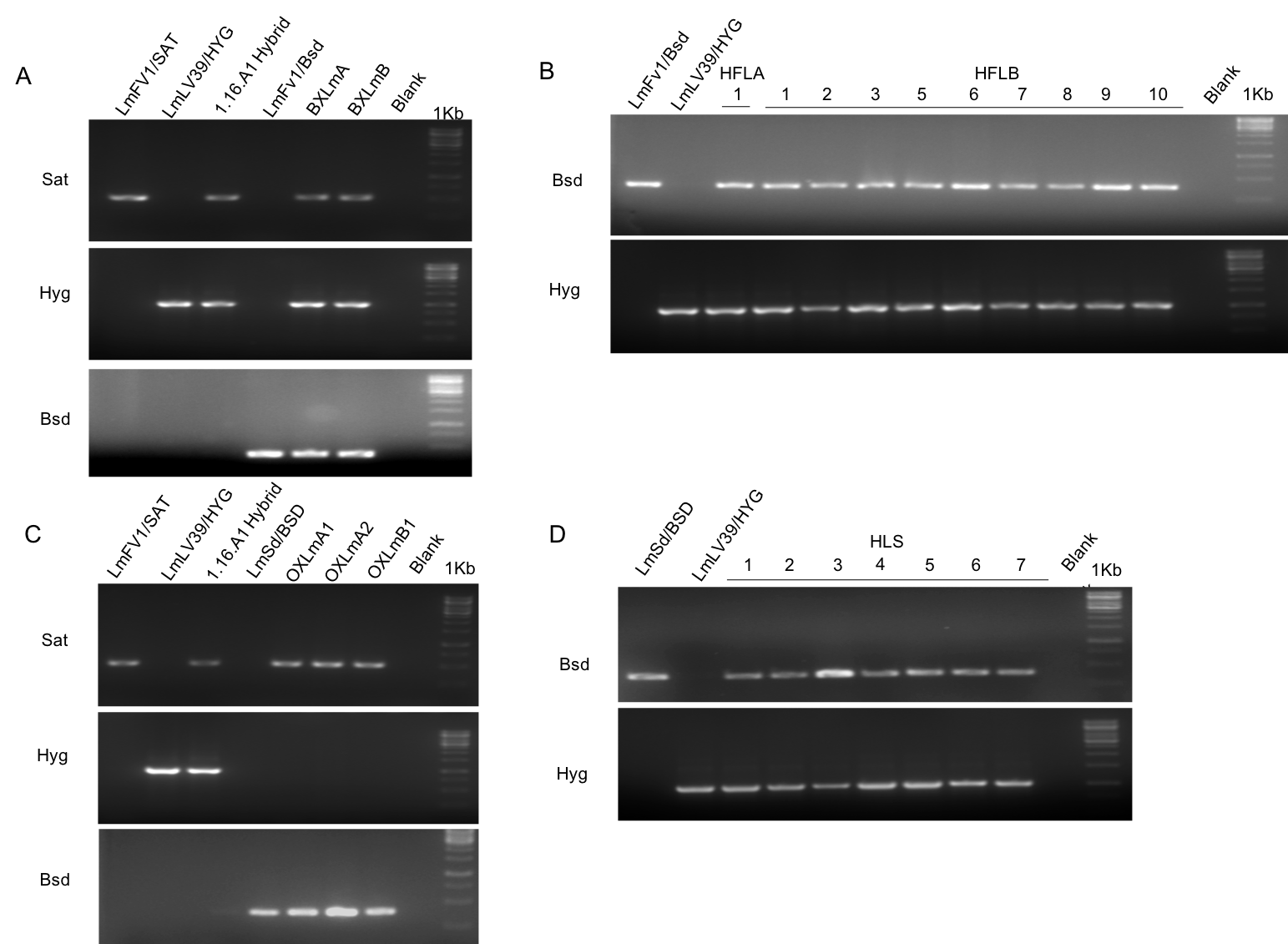

Supplement: S2 Fig — A) PCR for SAT, HYG and BSD resistance genes on the parental lines LmFv1/Sat, LmLV39/HYG, 1.16A1 –hybrid generated by these two lines, LmFv1/BSD and two backcross lines, BXLmA and BXLmB generated by crossing LmFv1/BSD and 1.16.A1. B) PCR for HYG and SAT on the parental lines LmFv1/BSD and LmLV39/HYG, and 10 hybrids generated by crossing these two lines (HFL) in two different experiments (A and B). C) PCR for SAT, HYG and BSD in 3 outcross lines generated in two experiments (LmOXA and LmOXB) in which 1.61.A1 hybrid was crossed with LmSd/BSD. D) PCR for HYG and BSD in 7 hybrids generated by crossing LmSd/BSD and LmLV39/HYG. Blank is PCR mix with no template. (PNG) [file pgen.1008042.s005.png]

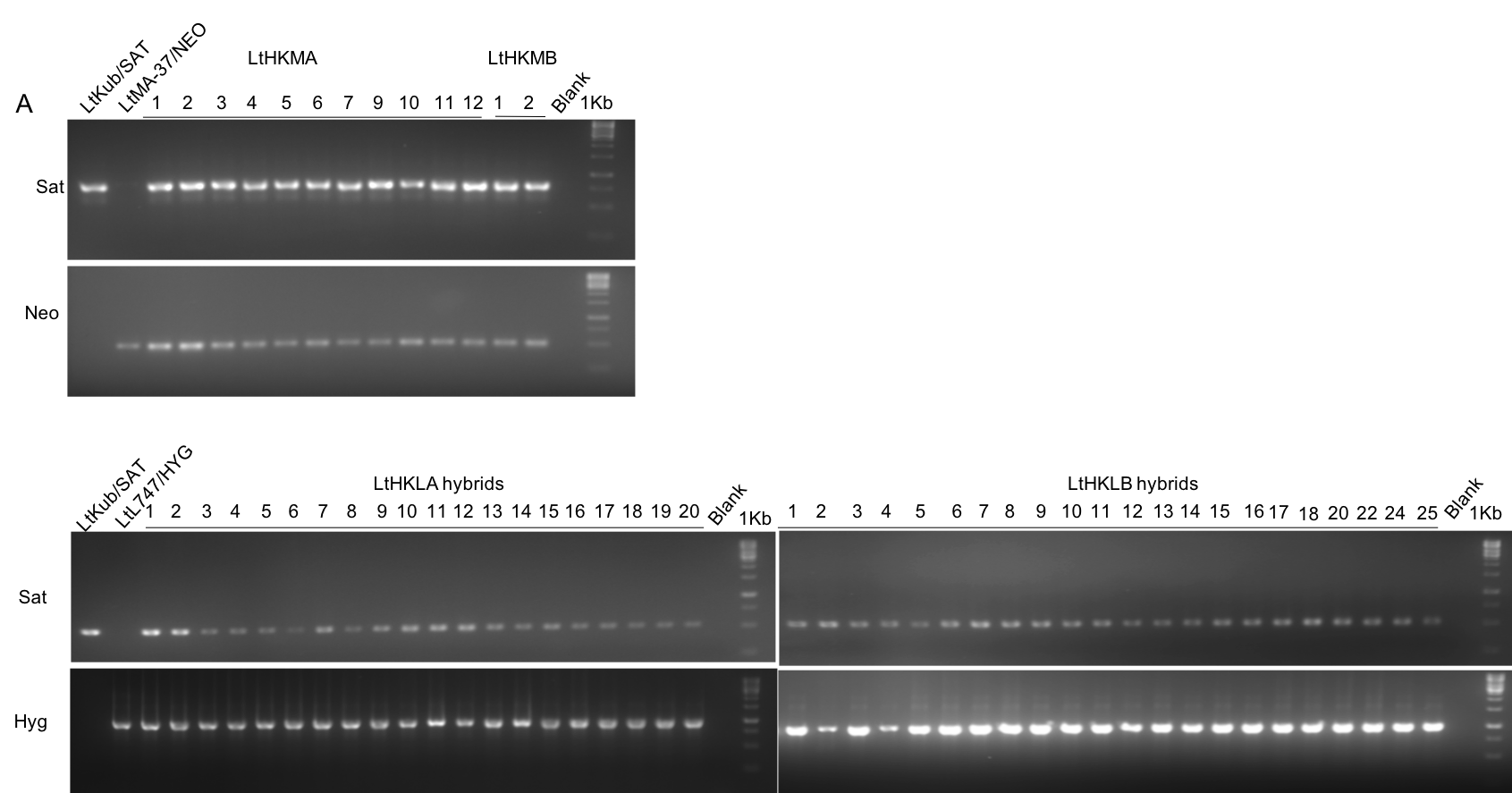

Supplement: S3 Fig — A) PCR on DNA extracted from the parental lines LtMA37/NEO and LtKub/SAT, and 13 hybrid clones (LtHKM), or the parental lines LtL747/HYG and LtKub/SAT, and 42 hybrid clones (LtHKL), generated in two experiments (A & B). Hybrids were recovered on selection medium containing SAT and NEO or SAT and HYG. Blank is PCR mix with no template. (PNG) [file pgen.1008042.s006.png]

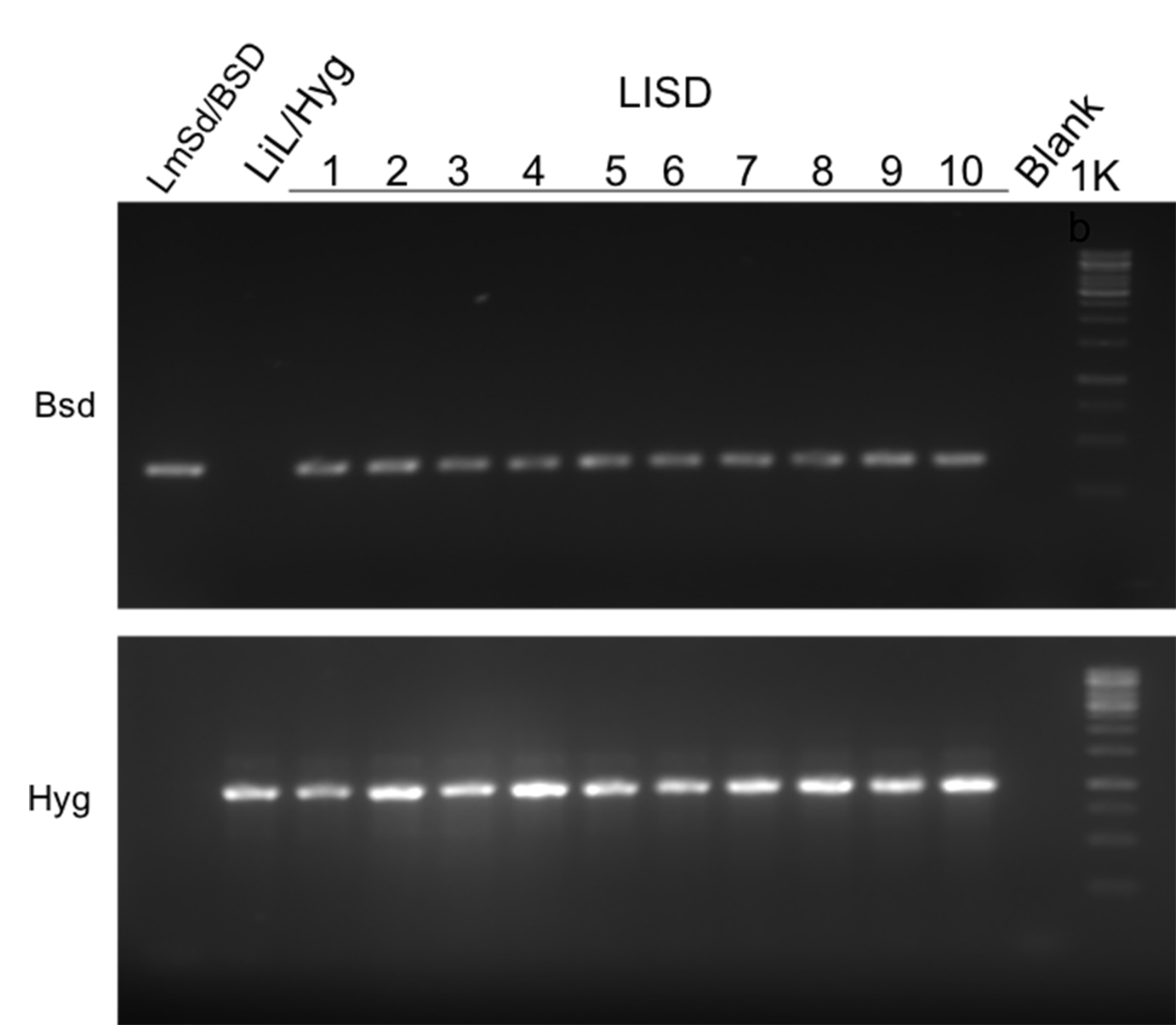

Supplement: S4 Fig — PCR for HYG and BSD on DNA extracted from the parental lines LmSd/BSD and LiL/HYG, and 10 hybrids generated by crossing these two lines. Blank is PCR mix with no template. (TIF) [file pgen.1008042.s007.tif]

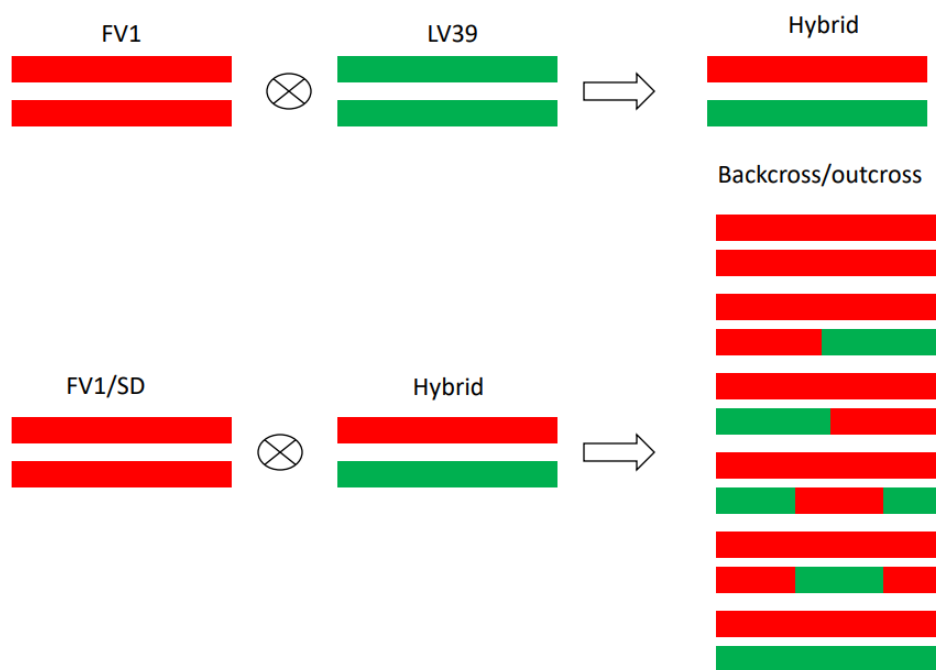

(A)

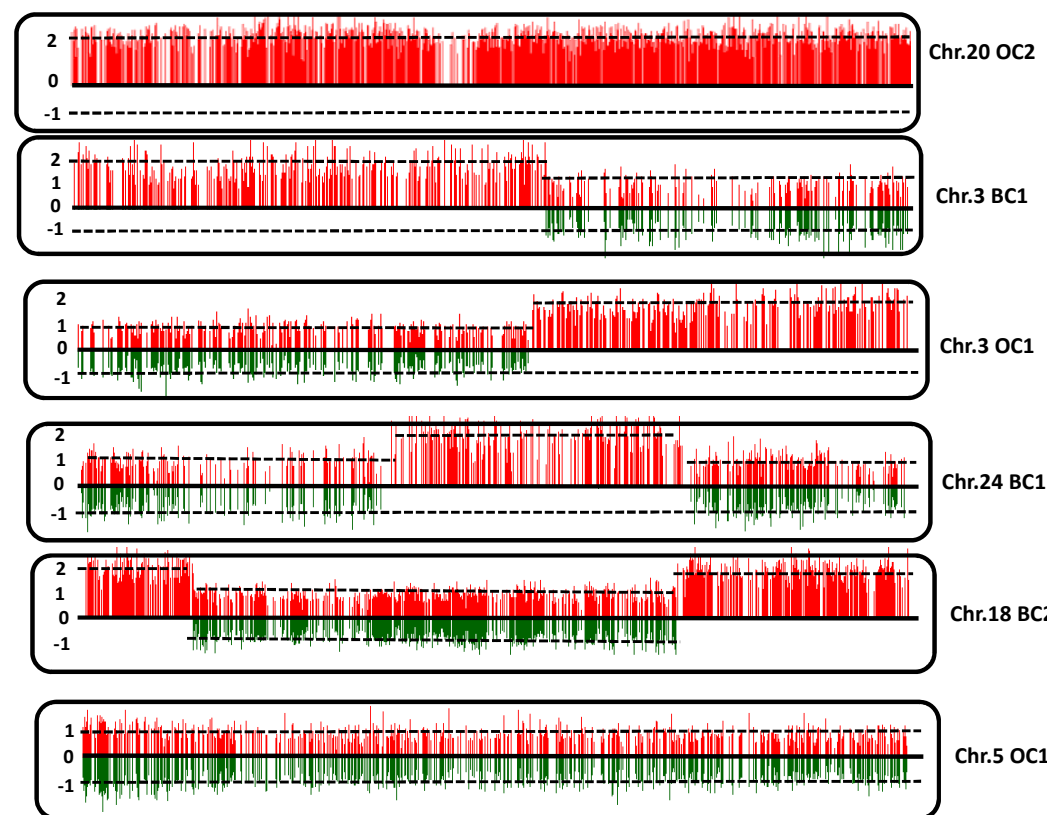

(B)

Supplement: S5 Fig — A) Schematic showing the possible recombinations assuming not more than two crossovers per chromosome. B) Bottle brush plots of representative chromosomes showing the possible inheritance patterns in the 5 hybrid clones generated between the F1 hybrid, 1.16.A1, and either LmFV1/BSD (BC1-2) or LmSd/BSD (OC1-3). SNPs inheritance from LmFV1 or LmSd are shown on the positive x-axis in red, and SNPs inheritance from LmLV39 are shown on the negative x-axis in green. The vertical distance corresponds to the inferred allelic depth, normalized across the entire genome, which was assigned an average somy of 2. (PDF) [file pgen.1008042.s008.pdf]

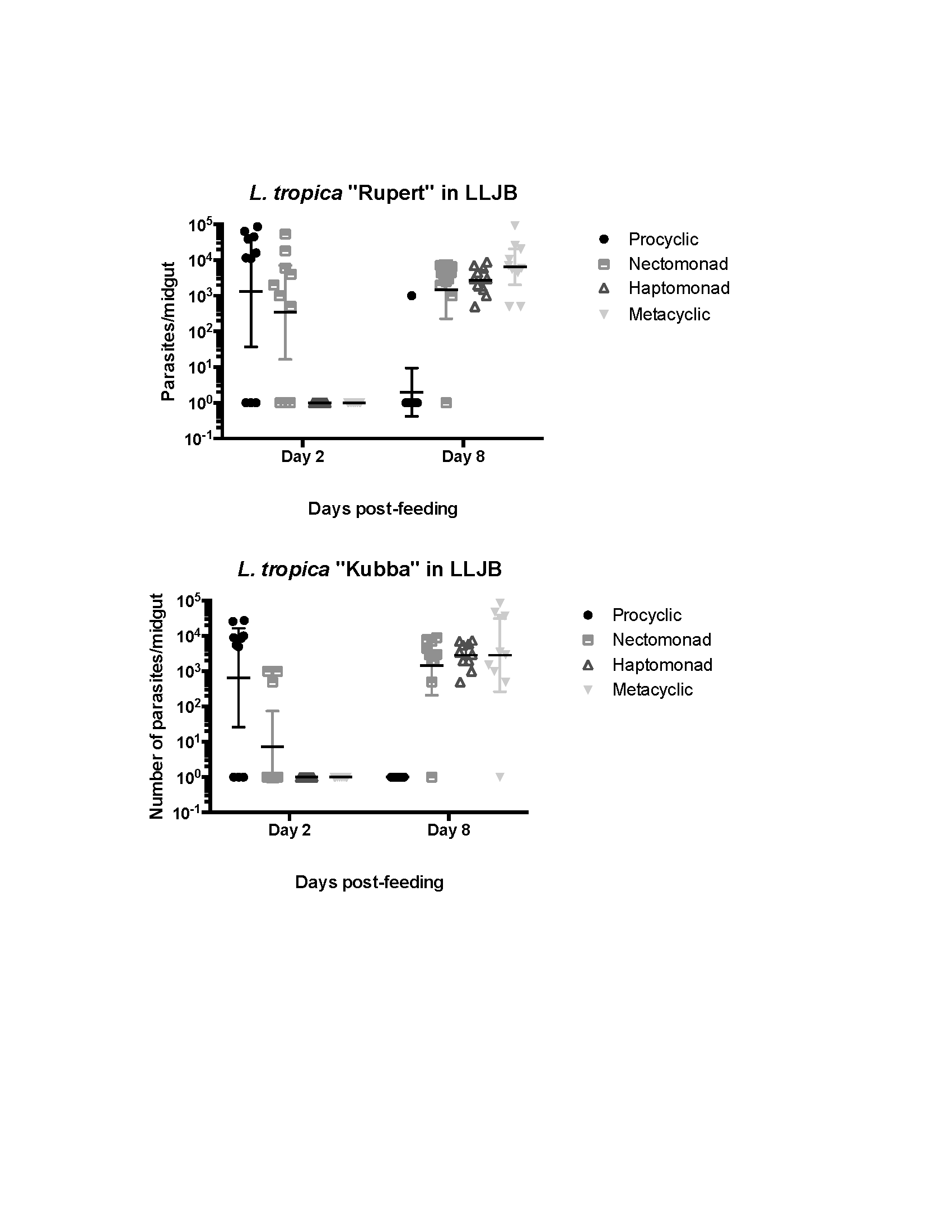

Supplement: S6 Fig — Flies were membrane fed on mouse blood containing with 4x10^6^ / ml of either LtRupert or LtKubba log phase promastigotes. Flies were dissected days 2 or 8 post-infection and homogenized midguts scored under a hemocytometer for the number of each of 4 different developmental stages of promastigotes. Values shown are individual flies with geometric means +/- 1 s.d., 10 flies / group at each time point. (TIFF) [file pgen.1008042.s009.tiff]

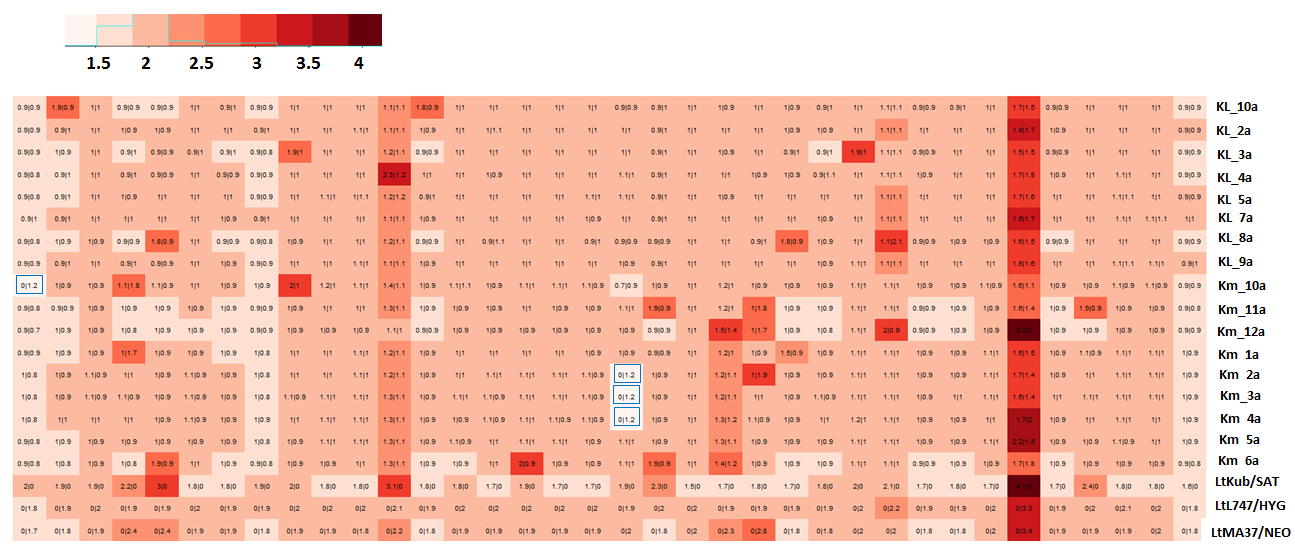

Supplement: S7 Fig — The read alignments to the genome were translated into somies and depicted as a heatmap, rounded off to the nearest 0.25 value. Overlaid are the parental inheritance values in the format LtKub-SAT/LtL747-HYG or LtKub-SAT/LtMA37-NEO, rounded off to the nearest 0.1 value. Monosomic chromosomes showing a single parent contribution are indicated by blue boxes. (TIF) [file pgen.1008042.s010.tif]
